# Materials Design and System Construction for Conventional and New‐Concept Supercapacitors

**DOI:** 10.1002/advs.201600382

**Published:** 2017-02-03

**Authors:** Zhong Wu, Lin Li, Jun‐min Yan, Xin‐bo Zhang

**Affiliations:** ^1^ State Key Laboratory of Rare Earth Resource UtilizationChangchun Institute of Applied ChemistryChinese Academy of SciencesChangchun130022China; ^2^ University of Chinese Academy of SciencesBeijing100049China; ^3^ Key Laboratory of Automobile MaterialsMinistry of Education and School of Materials Science and EngineeringJilin UniversityChangchun130012China

**Keywords:** integrated devices, material design, supercapacitors, system constructions

## Abstract

With the development of renewable energy and electrified transportation, electrochemical energy storage will be more urgent in the future. Supercapacitors have received extensive attention due to their high power density, fast charge and discharge rates, and long‐term cycling stability. During past five years, supercapacitors have been boomed benefited from the development of nanostructured materials synthesis and the promoted innovation of devices construction. In this review, we have summarized the current state‐of‐the‐art development on the fabrication of high‐performance supercapacitors. From the electrode material perspective, a variety of materials have been explored for advanced electrode materials with smart material‐design strategies such as carbonaceous materials, metal compounds and conducting polymers. Proper nanostructures are engineered to provide sufficient electroactive sites and enhance the kinetics of ion and electron transport. Besides, new‐concept supercapacitors have been developed for practical application. Microsupercapacitors and fiber supercapacitors have been explored for portable and compact electronic devices. Subsequently, we have introduced Li‐/Na‐ion supercapacitors composed of battery‐type electrodes and capacitor‐type electrode. Integrated energy devices are also explored by incorporating supercapacitors with energy conversion systems for sustainable energy storage. In brief, this review provides a comprehensive summary of recent progress on electrode materials design and burgeoning devices constructions for high‐performance supercapacitors.

## 1 Introduction

The past decades have witnessed the rapid depletion of fossil fuels and increasingly worsened environmental pollution, which make energy systems are one of the most important topics in future.^1^, ^2^, ^3^ It has received increasing research interest to seek highly efficient, low cost, and environmentally benign energy sources to substitute fossil fuels.^4^ Due to ever‐increasing human reliance on energy‐based appliances, electrical energy has become the most important secondary energy source and is indispensable in aspects of daily life.^5^ Therefore, the search for the next generation energy storage devices with efficient use of energy is extremely important.^6^ Storage devices with high energy and power densities are in strong demand for most systems such as cellular phones, electric cars, and so on.^7^ Among various energy storage systems, supercapacitors (SCs) and lithium batteries are at the frontier of this research.^8^, ^9^ SCs and battery devices as representative modern energy storage devices have been widely used in our daily life by powering various portable electronic devices and even current plug‐in hybrid electric vehicles.^10^, ^11^

**Figure 1:**
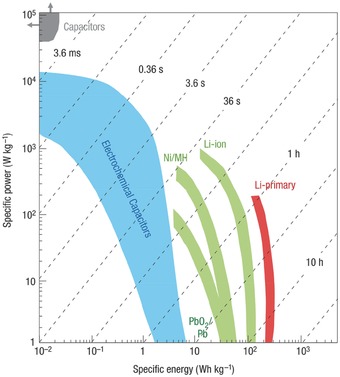
Ragone plot showing specific power against specific energy output for SCs and batteries devices. Reproduced with permission.^12^ Copyright 2008, Nature Publishing Group.

**Figure** **1**, 12 presents the simplified ‘Ragone plot’ of various energy conversion and storage devices according to their specific power and specific energy output. In comparison, SCs occupy an important position in terms of the specific energy as well as the specific power. Firstly, compared with traditional electrolytic or electrostatic capacitors, SCs can deliver much higher energy density due to the charge storage in an electric double layer in place of a dielectric layer.^13^ Secondly, SCs can provide faster power delivery than lithium batteries.^14^, ^15^ High power density (>10 kW kg^–1^) means the system can release and deliver high current or uptake pulse when is required.^16^ SCs are one of the crucial power devices that can be fully charged/discharged in seconds. Hence, SCs are well‐suited for regenerative braking, frequency regulation in smart grids and storing the intermittent energy profiles of renewable energy sources.^17^, ^18^ Currently, SCs are being envisaged for several applications in consumer electronics, memory back‐up systems, industrial power and energy management.^19^, ^20^ To some extent, SCs fill the gap between conventional capacitors and lithium batteries. However, lithium batteries can deliver higher energy density reaching 200 Wh kg^–1^ whereas SCs show an energy density in the range of 5–10 Wh kg^–1^.^21^ In brief, SCs possess superior power density but limited energy density compared to lithium batteries.

The biggest challenge for SCs research is to enhance their specific energy density while retain their intrinsic high specific power density. To date, a great deal of research efforts has been devoted on increasing the energy performance of SCs to be close to or even more than that of batteries. This review begins with the typical in‐depth study on the charge storage mechanism and then illustrates current state‐of‐the‐art improvement in SCs regarding the electrode materials design and booming devices construction. On the following sections, the characteristics will be discussed in details for SCs based on different charge storage mechanisms. Progress towards supercapacitor technologies can make an achievement from the fundamental understanding of charge/discharge behavior of SCs. Besides, SCs is potential for large‐scale deployment such as industrial equipment market and solar electricity storage.

## 2 Charge Mechanism

SCs also known as ultracapacitors and electrochemical capacitors have attracted intense attentions due to their high specific power, a long cycle life, and fast charge/discharge processes (within seconds).^22^ Generally, SCs can be divided into electrical double layer capacitors (EDLCs) and pseudocapacitors (PCs) depending on their energy storage mechanism.^23^ EDLCs store the charge through the ion adsorption/desorption on the electrode/electrolyte interfaces. EDLCs mainly based on high surface area carbon materials which are electrochemically stable. Charge storage in PCs depends on the fast reversible surface redox reactions. Several metal compounds and conducting polymers are the typical materials used for PCs such as RuO_2_,^24^ MnO_2_,^25^ polypyrrole,^26^ polyaniline,^27^ and polythiophene.^28^ The mechanisms occur mainly on the electrode/electrolyte interface, which ensure SCs fast charge and discharge rates and high power density, and these two mechanisms can occur simultaneously in some cases.

### 2.1 Mechanism of EDLCs

EDLCs store energy through the ion absorption and desorption during the charge/discharge processes on the electrode/electrolyte interface without the charge transfer reaction.^29^, ^30^ The capacitive behaviors are strongly dependent on the surface area of the electrode materials that is accessible to the electrolyte ions.^31^, ^32^ It can be expressed as follows:

(1) $$C\;=\;\frac{\varepsilon_{r}\varepsilon_{0}A}{d}$$

where *C* is the capacitance of the EDLCs, ε_r_ is the electrolyte dielectric constant, ε_0_ is vacuum permittivity (8.854 × 10^–12^ F m^–1^), *d* is the effective thickness of the double layer located at the electrolyte/electrode interfaces, and *A* is the electrode surface area immersed in the electrolyte. Since *d* in EDLCs is just a few angstroms, which is far less than it in traditional dielectric capacitors.^33^, ^34^ hence, higher capacitance can be achieved in EDLCs. The capacitance of EDLCs is dependent on the specific surface area of the electrode, the type of electrolyte and the effective thickness of the double layer.

During the charging process, anions of electrolytes are accumulated on the positive surface of EDLCs, while the surface of negative electrode attracts the cations of electrolytes.^35^, ^36^ The energy storage process involves highly reversible non‐faradaic reactions occurring at the electrode/electrolyte interface. This storage mechanism of EDLCs allows for very fast energy uptake and delivery and long cycling life with almost no capacitive fading as compared with other energy storage systems. The underlying cause may be attributed to the physical adsorption and desorption process.

To obtain superior capacitance, it is required to optimize the pore size, pore structure, surface properties and conductivity of the electrode materials.^37^ Carbon‐based materials are used as EDLCs electrodes due to their high surface area and electronic conductivity. Capacitance values of many tens of farads per gram of the electrode material are achieved in some materials, such as activated carbon, graphene, carbon nanotube, and so on.^38^, ^39^, ^40^ Nevertheless, the electrical double layer capacitance derived from the charge separation is still not able to meet the commercial market expectation in terms of their limited specific energy density.^41^

### 2.2 Mechanism of EDLCs

**Figure 2:**
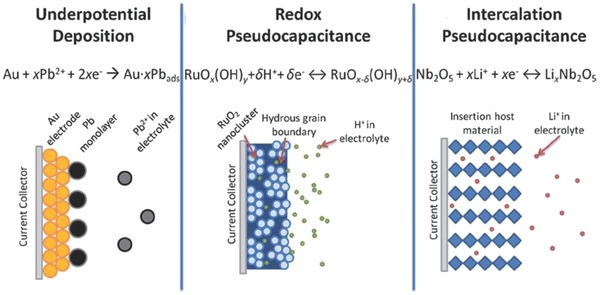
Different types of reversible redox mechanisms that give rise to pseudocapacitance: underpotential deposition, redox pseudocapacitance, and intercalation pseudocapacitance. Reproduced with permission.^46^ Copyright 2014, RSC.

Unlike the EDLCs that physically accumulates charges, pseudocapacitance is a faradaic process that involves surface or near‐surface redox reactions.^42^ According to Conway's book,^43^ charge transfer process of PCs that exploits the faradaic reaction to tunnel the electrons across the current collector/electrode^44^, ^45^ interfaces can be identified as three faradaic mechanisms as followings, which is illustrated in **Figure** **2**, 46: (1) underpotential deposition (UPD), (2) redox pseudocapacitance, (3) intercalation pseudocapacitance.

UPD involves the faradaic absorption/desorption of metal ions or protons on the surface of metallic active materials (e.g. Au, Pt, Rh, and Ru) to form an adsorbed monolayer above their redox potential, thus UPD is also called two‐dimensional adsorption pseudocapacitance.^47^, ^48^

Redox pseudocapacitance occurs at a surface or near surface of electrode material when ions are electrochemically adsorbed, accompanied by the charge transfer process.^49^ Recently various active materials have been widely investigated as the potential candidates to exhibit redox pseudocapacitance, such as such as transition metal oxides (RuO_2_,^50^ MnO_2_ 51) and conducting polymers (polyaniline,^52^ polypyrrole^53^). During charging/discharging, transient change in valence states of such active materials occurs with concomitant faradaic charge transfer.

Intercalation pseudocapacitance refers such a process that cations intercalate into the tunnels or layers of host materials accompanied by a faradaic charge‐transfer involving no crystallographic phase change.^54^ It should be noted that two‐dimensional pathway for ion transportation with minimum structural distortion is needed for this behavior. Materials with the rigid layered lattice framework are require, such as Nb_2_O_5_,^55^ V_2_O_5_,^56^ and MoO_3_.^57^ The difference for three mechanisms is their respective different physical processes and the similarity is their electrochemical signatures.

PCs typically exhibit higher specific capacitance and energy density than EDLCs. In general, the specific capacitance associated with faradaic reactions is at least 10 times greater than that of double‐layer processes.^58^ However, PCs often suffer from low power density and lack of stability due to poor electrical conductivity and framework swelling during cycling.^59^ The electrode materials should possess redox capability to maintain the charge balance by chemisorption of electrolyte ions upon the continuous charge/discharge processes.

### 2.3 Electrochemical Characterization

There are many parameters of defining the overall electrochemical performance of SCs in terms of specific capacitance, operating voltage, equivalent series resistance, power density, energy density, and time constant.^60^, ^61^ To accurately assessed the capacitive behaviours, a variety of methods have been proposed to measure the three essential parameters: specific capacitance (*C*_s_), operating voltage (*V*) and equivalent series resistance (ESR). And then power density (P) and energy density (*E*) can be calculated based on the three essential parameters. The common methods for measuring the three fundamental parameters include cyclic voltammetry (CV), galvanic charge/discharge (GCD) and electrochemical impedance spectroscopy (EIS).

Firstly, CV testing can be used for examining the charge storage mechanism and calculating the specific capacitance of the electrode materials in a three‐electrode configuration. CV curves can be obtained based on their response to a voltage sweep which is plotted as current vs. potential. In EDLCs, an almost rectangular is observed in CV curves resulted from the highly reversible adsorption and desorption of ion at electrode‐electrolyte interface.^62^ The CV curves of PCs can present a rectangular or redox couple peaks resulted from the redox reactions.^63^, ^64^, ^65^

Specific capacitances (F g^–1^) of the electrode materials can be calculated based on CV curves according to the following equations^43^:

(2) $$Cs\;=\;\frac{\int OIdV}{2m\nu \Delta V}$$

Where *I* (mA) is the instantaneous current, Δ*V* (V) is the applied potential window which presents the range of potential change, *m* (g) is the weight of the active material, and *v* (mV s^–1^) is the scan rate which presents the speed of the potential change during the positive and negative sweeps in the CV measurement.

Secondly, GCD testing is used for the characterization of capacitive behaviours based on their response to the constant current. GCD curves can be obtained according to the functional relation between the potential and time. In a three‐electrode configuration, the specific capacitance can be calculated based on GCD curves according to the following equations^43^:

(3) $$Cs\;=\;\frac{I}{mdV/dt}$$

Where *I* (A) is the discharge current, *m* (g) indicates the mass of the active material, and the value of d*V* (V)/d*t* (s) represents slope obtained from the discharge curve in the GCD measurement. In the case of nonlinear response for most of the pseudocapacitive materials, it is suggested to calculate the specific capacitance using the two datum points derived from the discharge curve, d*V*/d*t* = (*V*_max_–1/2 *V*_max_)/(*t*_2_ – *t*_1_), where *t*_1_ and *t*_2_ represent the discharge time of *V*_max_ and 1/2 *V*_max_, respectively.

Thirdly, EIS is always conducted to investigate the transport characteristics of the charge carriers within the capacitive electrode.^66^, ^67^ EIS measures the impedance of the devices as a function of frequency by applying a low‐amplitude alternative voltage superimposed on a steady‐state potential, which are usually expressed graphically in a Nyquist plot. In a Nyquist plot, the expression of impedance (Z) can be simplified as: Z = Z′ + jZ′′, in which Z′ and Z′′ could be defined as the real and imaginary part of impedance, respectively. By executing similar analysis, the interpretation of the impedance results can be used to probe aspects of capacitive behaviors of the electrode materials including specific capacitance, charge transfer, mass transport and charge storage mechanisms.^68^, ^69^

Furthermore, energy density (*E*) and power density (*P*) are two important parameters to evaluate the performance of energy storage devices. Energy density represents the total amount of charges stored in SCs per unit mass or volume, whereas the power density is synonymous to the rate of charge being delivered upon discharging. The equations used to express energy density and power density are as follows^43^:

(4) $$E\;=\;\frac{1}{2}CV^{2}$$

(5) $$P\;=\;\frac{V^{2}}{4mR_{S}}$$

Where *E* (J g^−1^) is energy density, *C* (F g^–1^) is the specific capacitance of the electrode material, *V* (V) is the potential range, *m* (g) is the mass of electrodes, and *R_s_* is the equivalent series resistance (ESR) in ohms. Here, the ESR is deduced from the following equation^43^:

(6) $$ESR\;=\;\frac{\Delta V}{\left|I_{ch\arg e}\right|\;+\;\left|I_{disch\arg e}\right|}$$

Where △*V* represents the voltage different. *I*_charge_ and *I*_discharge_ represent magnitude of the charge and discharge currents, respectively. According to the energy density formula, the enhanced energy density can be obtained by increasing the specific capacitances and/or widening the potential range.^70^, ^71^

Besides, long‐term cycling life is crucial for an electrode material to be used in electrochemical capacitors and their further practical application. Cycling stability can be obtained by observing the degradation before and after a long‐term cycling process through CV or GCD measurements.

### 2.4 Electrochemical Testing System

**Figure 3:**
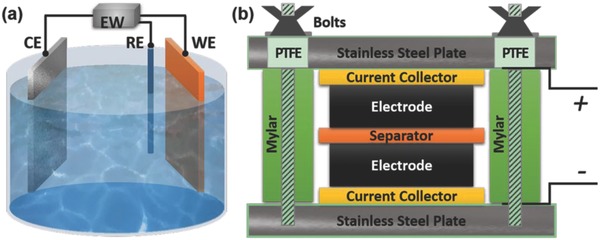
Construction graphs of (a) the three‐electrode testing system (EW: electrochemical workstation, WE: working electrode, CE: counter electrode, RE: reference electrode), and (b) the two‐electrode testing system.

Generally, the electrochemical properties of electrode materials are evaluated in a three‐electrode or two‐electrode systems. The three‐electrode testing system is composited by working electrode (WE), counter electrode (CE) and reference electrode (RE), which is illustrated in **Figure** **3**a. Three‐electrode testing system is always employed to investigate the electrochemical performances of the active materials. In a three‐electrode setup, the applied voltage across the working electrode is measured with respect to a particular reference electrode.^72^

Nonetheless, the two‐electrode testing system is composed by a pair of closely spaced electrodes containing the electro‐active materials attached to the current collector saturated with an electrolyte and a separator sandwiched between the parallel electrodes, as displayed in Figure 3b.^73^ The separator is a dielectric porous membrane with ion permeable but electrical insulating that allows migration of electrolyte ions while keeping the two electrodes electrically apart. On one hand, the separator acts as a physical barrier between the two electrodes to prevent electrical short circuits. On the other hand, the separator is permeable to the ions allowing rapid ionic charge transfer during the passage of current in an electrochemical cell.

For two‐electrode testing system, the whole electrochemical performance of SCs mainly depends upon both the two electrodes in the given electrolytes. The whole construction is a cell, and the two electrodes are positive and negative electrodes, respectively. In terms of two‐electrode configuration, an SCs cell are grouped into two major categories called symmetric and asymmetric devices depending on the kinds of electrode materials.^74^

In the case of the symmetric cells, these devices will be composed using the same material with the same mass for both the positive and negative electrodes. In symmetric SCs, the applied voltage is distributed equally on two electrodes. Such a symmetrically configured cell has both electrical double layer capacitance and pseudo‐capacitance. Symmetric capacitors are EDLCs‐based devices, and they store electrostatic charge from the accumulation and separation of ions at the interface between the electrode surface and electrolyte. On this occasion, aqueous, organic or ionic electrolyte solutions are flexible at the request of the existing system.^75^ Another symmetric capacitors is constructed of pseudocapacitive materials with reasonable potential range. Manganese dioxide‐based electro‐active materials have been investigated in pseudocapacitive symmetric capacitors.^76^ It should be noted that this type of symmetric SCs is rarely used since the redox reactions only occur either at the positive or the negative voltage, but not on both.

In the case of asymmetric SCs, one pseudocapacitive electrode and one EDLCs electrode have been used for positive and negative electrodes, respectively. In general, the asymmetric devices feature a high energy density and a high power density due to an increased operating potential.^77^ Asymmetric SCs would combine the advantages of different electrode materials and then achieve a wider operating voltage for the device. By using different types of electrodes that can be operated at different potential windows, an asymmetric capacitor can be manufactured with cell voltages extending up to 2.0 V in an aqueous electrolyte.^78^ To improve asymmetric SCs performance, effective methods involve the advanced development of electrode materials with optimized architectures. It is worth noting that the matching ratio of positive and negative electrodes should be considered for smart SCs configurations.^79^ The different mass ratio between positive and negative electrodes will affect the overall capacitance of the device.^80^, ^81^ Therefore, it is important to optimize the mass ratio between positive and negative electrodes in two‐electrode system.

In brief, the electrochemical performances of SCs are governed by several parameters including the active electrode material, the nature of electrolyte, and the testing systems. Usually, the effects of these parameters are interlinked and revealed in combination.

## 3 Electrode Materials

Electrode material is an important factor determining the specific capacitance, which has been receiving intensive attention from research.^82^ It is evident that controlling the architecture and interface property of electrode materials plays an important role in enhancing high‐performance SCs including high specific capacitance, good rate capability and long cycling stability.^83^ Fortunately, nanotechnology has opened up new frontiers in materials science and engineering and will play a critical role in advancing the development of charge storage to meet the challenge.^84^ The morphology and chemical composition of the resultant materials are characterized by scanning electron microscopy (SEM), transmission electron microscopy (TEM), scanning transmission electron microscopy (STEM), powder X‐ray diffraction (XRD), energy dispersive X‐ray spectroscopy (EDX), and X‐ray photoelectron spectroscopy (XPS) measurements and so on. Over the past decades, technological breakthrough in nanoscience paves a new way for the development of materials design, and the electrochemical performance is boosted to several‐fold capacitive enhancement.

Despite of different mechanisms based on EDLCs and PCs, there are some common traits for enhanced electrochemical properties. An electrode material with high surface area and high electric conductivity is expected to develop advancement for SCs.^85^ At first, high surface area can provide more active sites for EDLCs or pseudocapacitive reactions since the increased surface‐to‐volume ratio supplies maximum contact surface between the active materials and electrolyte ion. High surface area can be obtained by synthesizing nanosized or/and porous electroactive materials.^86^ In this case, porous structure for electrolyte diffusion is helpful to guarantee easy accessibility for ion penetration, avoiding sacrifices of surface area due to stacking or blockage of active sites. Secondly, high electrical conductivity for fast charge transportation is crucial to realize a high rate capability and power density. The enhanced electric conductivity can be realized by decreasing the size of the electroactive material and/or compositing with other materials with good electrical conductivity.^87^

The performances for an electrode material are investigated by assembling them into a three‐electrode or two‐electrode systems. In traditional equipment for an electrode, the electrode material, conductive agent, and binders are grinded to form uniform slurry and then it is pasted to current collectors.^88^ Conductive agents are conductive carbon (e.g. acetylene black) and used to improve the electrical conductivity of the electrode. The presence of binders helps the active materials and conductive agent to be linked and make them good mechanical adhesion to the current collector. However, binders are generally electro‐inert, which bring about potentially greater internal resistance. So far, flexible electrode has emerged as a new electrode configuration with mechanically robust achieving high specific gravimetric and volumetric capacitance. Flexible electrodes have been designed by self‐standing electrode materials with high flexibility or integrated electrode materials with high flexibility on soft‐matter substrates, wherein conductive agent and binder are unnecessary.^89^, ^90^ In this part, potential flexible electrode materials electrode will be briefly introduced.

### 3.1 Carbon Materials

Carbon materials are generally carbon‐based materials, which store charges electrostatically at their surfaces using reversible adsorption/desorption of ions of the electrolyte onto active materials.^91^ Carbon‐based materials have been widely explored as electrode materials for SCs due to a unique combination of properties such as high surface area, lightweight, good electrical conductivity, controlled pore size distribution and compatibility with other materials. The carbon‐based electrochemical capacitor is first discovered by Becker in 1957. Activated carbon electrodes have been applied into commercial SCs with high power density, yet their energy density is limited which is mainly due to the ill‐defined physical and chemical properties of the activated carbon.^92^ More research has focused on developing high‐performance SCs based on carbon materials with low‐cost, versatile, highly conductive, chemically stable at wide temperature range. Additionally, carbon‐based free‐standing electrodes are promising for producing wearable electrodes due to their light weight, flexibility, and highly conductive.^93^, ^94^

#### 3.1.1 Graphene

Graphene, a two‐dimensional carbon sheet with monoatomic layer thickness, has been widely explored as an ideal electrode material for SCs in the past decade.^95^, ^96^ Graphene features unique properties including high theoretical surface area (2630 m^2^ g^–1^) and high in‐plane electrical conductivity.^97^ Graphene sheets (single layer to a few layers)have been synthesized by several effective and facile approaches such as mechanical exfoliation method,^98^ exfoliation of graphite in organic solvents,^99^ epitaxial growth and chemical vapor deposition (CVD),^100^ and the exfoliation and reduction of chemically oxidized graphite.^101^, ^102^ Mechanical exfoliation (or the “scotch tape” method) can obtain single graphene but is difficult to massively produce graphene for the practical application.^103^

**Figure 4:**
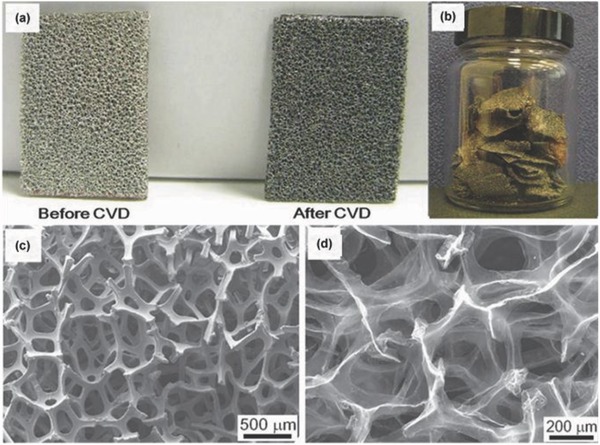
a) Photographs of Ni foam before and after the growth of graphene. b) Photograph of the obtained graphene in a single CVD process after removal of Ni foam.c) SEM image of graphene on Ni foam after CVD, and d) SEM image of graphene networks after removal of Ni foam. Reproduced with permission.^109^

Chemical vapor deposition (CVD) on a metal substrate (Ni, Cu, etc.) has been recognized as an alternative method for fabrication of graphene sheets with high conductivity comparable to the pristine graphene.^104^ The as‐synthesized graphene possess superior electrical conductivity and high surface area which would facilitate the fast electron transport between the active materials and current collectors.^105^ Kong et al. first reported the CVD growth of few‐layer graphene films on the polycrystalline Ni substrate.^106^ However, the employed carbon sources are usually explosive (e.g. CH_4_).^107^, ^108^ Alternative substrates and safe carbon sources for graphene growth have been developed in recent years for mass production. Zhang et al. have reported a good method to scale up the CVD synthesis of graphene by using Ni foam as a sacrificial template and ethanol as the carbon source.^109^ As illustrated in **Figure** **4**a, the colour of the Ni foam changed from shiny white to dark gray after the growth of graphene. Figure 4b shows the yield of graphene, the production can be easily scaled up by using a larger CVD chamber. Ethanol used as carbon sources are safe and cheap, which is essential for practical applications. Figure 4c and d investigated the three‐dimensional graphene networks grown on Ni foam after the CVD process and three‐dimensional graphene networks after the removal of Ni foam. Additionally, the as‐synthesized graphene networks can serve as templates for the construction of graphene/NiO composites. Superior electrochemical properties are demonstrated containing a high specific capacitance of 816 F g^–1^ and a stable cycling performance without any decrease after 2000 cycles.

Among these synthesis methods, the exfoliation and reduction of chemically oxidized graphite is used for large‐scale production of graphene.^110^ A considerable amount of researches have been stimulated on developing cost‐effective graphene‐based energy storage materials with enhanced energy and power density as well as long life‐cycle.^111^ In this case, graphite is oxidized and exfoliated into graphite oxide (GO) and then graphene is prepared after an reduction process.

GO can be synthesized by either the Brodie,^112^ Staudenmaier,^113^ or Hummers^114^ methods at large‐scale with relatively low cost. The as‐synthesized GO contains oxygen‐containing functional groups including epoxide, carbonyl, hydroxide and carboxylic acid groups. GO has been used as SCs electrode material and exhibits a higher capacitance (189 F g^–1^) than that of graphene due to an additional pseudocapacitance effect resulted from the oxygen‐containing functional groups on its basal planes.^115^ Deng et al. found that the specific capacitance increases when the oxygenated species reduces.^116^ Additionally, GO is easy to be modified for the fabrication of high‐performance SCs.^117^ Kim et al. have reported progress toward high‐performance SCs based on poly(ionic liquid)‐modified GO electrodes via electrostatic interactions.^118^ Stable electrochemical performance with a high specific capacitance of 187 F g^–1^ was observed due to enhanced compatibility with certain ionic liquid electrolytes and improved accessibility of electrolyte ions.

Various routes have been used to reduce GO to graphene to restore the intrinsic specific surface area and electrical conductivity of graphene, and construct graphene‐based nanostructures with desirable channel size, which are all favourable for SCs applications.^119^, ^120^ GO can be reduced by reducing aqueous solutions such as hydrazine hydrate, hydrobromic/hydroiodic acid, and so on.^121^ However, the sheet‐to‐sheet van der Waals interactions between graphene sheets lead to the stacking of the graphene sheets. Consequently, the specific surface area is reduced during the stacking process and inferior specific capacitance is observed.^122^ Hence, extensive approaches have been explored to reduce the aggregation between graphene sheets.

Firstly, surfactant intercalation has been regarded as a facile method to inhibit aggregation of graphene sheets during reduction. Zhang et al. have conducted schematic studies on a series of surfactant‐stabilized graphene materials with different surfactants.^123^ The presence of surfactants benefits to stabilize the morphology of single layer or few‐layer structure of graphene sheets during reduction, and enable good dispersibility in aqueous solvents.

Secondly, thermal reduction is used for preparation of graphene to efficiently combine of electrically conductive channels and appropriate pseudocapacitive functional groups in the graphene‐based electrode at the same time.^124^ Zhi et al. have developed a striking strategy for elaborately constructing rationally functionalized graphene from natural graphite via a modified Hummers method followed by an acid‐assisted ultrarapid thermal‐processing technique.^125^ On one hand, ultrarapid thermal‐processing allows the efficient recovery of the basal structure electrically conductive channels. On the other hand, the ultrarapid thermal‐processing technique guarantees the preservation of functional groups with pseudocapacitive effects.

Thirdly, graphene sheets are assembled into three‐dimensional porous network and macrostructures.^126^, ^127^ To minimize the stacking of graphene sheets, other carbonaceous materials have been added between graphene sheets to design three‐dimensional materials with extraordinary properties. Taking carbon spheres as example, carbon spheres are used as spacer to separate graphene sheets by an assembly approach.^128^, ^129^ Electrochemical tests reveal enhanced cycleability and rate performance due to the synergistic effect of the combination of these materials.

**Figure 5:**
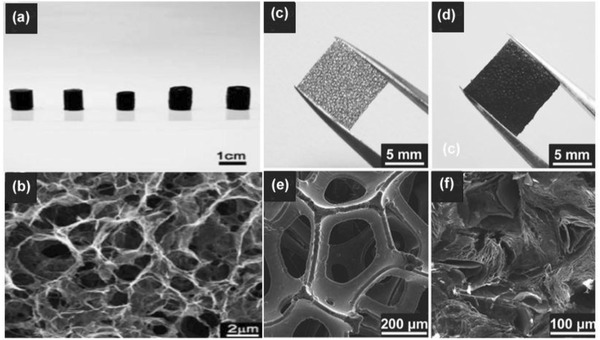
a) Photographs of graphene hydrogels with tunable height. b) SEM image of the interior microstructures of freeze‐dried graphene hydrogel. Reproduced with permission.^132^ Copyright 2010, ACS. c,d) Photographs of a piece of Ni foam before and after coating of graphene gel. e,f) SEM images of Ni foam before and after coating of graphene gel. Reproduced with permission.^134^

Graphene gels (hydrogels and aerogels) have received particular attention due to their lightweight, large specific surface area and interconnected three‐dimensional porous frameworks. Graphene gels allow multidimensional electron transport and rapid electrolyte ions diffusion.^130^, ^131^ For example, Shi groups have reported their works on graphene hydrogels as electrodes with high performance. At first, they prepared graphene hydrogels by a one‐step hydrothermal process as shown in **Figure** **5**a and b.^132^ The as‐prepared hydrogels possess a well‐defined three‐dimensional porous network. The unique three‐dimensional morphology provides open channels allowing graphene sheets exposed to electrolytes to optimize the ionic diffusion. The high performances of graphene‐base hydrogels make it promising for SCs applications. However, their capacitance decreases greatly at a high discharge rate and instability for long cycle life because of the relatively low conductivity and large amount of residual oxygenated groups of the hydrogel. To modify the electrochemical properties of graphene‐based hydrogels, Shi and co‐workers have reduced above‐mentioned graphene hydrogels with hydrazine or hydroiodic acid to improve their conductivities and remove their residual oxygenatedgroups.^133^ The as‐prepared reduced graphene hydrogels perform a high capacitance of 222 F g^–1^ at a low discharge rate and the capacitance can be retained 74% at an extraordinary fast discharge rate of 100 A g^–1^.

To further improve the capacitive performances of graphene hydrogels, one piece electrode has been constructed by in situ gelation of graphene sheets in the micropore of nickel foams as illustrated in Figure 5c–f.^134^ The porous structures and good conductivity of nickel foam lead to the shortened distance and fast response for charge transfer. Consequently, excellent performances are achieved including high specific capacitances, long durability, and high rate capability. In addition, Maiti et al. have demonstrated the scalable three‐dimensional shape‐engineering gelation of graphene by a simple immersion of zinc substrates.^135^ The thickness of graphene gel can be controlled by tuning the immersion time. The gel‐based SCs exhibit excellent rate capability due to the high electrical conductivity and facile ion transport compatible with large areal capacity resulted from constructing thick electrodes.

More approaches have been developed to construct three‐dimensional graphene to hold well‐defined pathways for efficient ionic and electronic transport.^136^ For example, Huh et al. build three‐dimensional graphene films by using polystyrene colloidal particles as a sacrificial template.^137^ Zhang et al. obtained porous three‐dimensional graphene‐based bulk materials through efficient and industrially scalable approach.^138^ The three‐dimensional precursor materials are prepared by in situ hydrothermal polymerization/carbonization of the mixture of cheap biomass or industry carbon sources with GO and then activated to achieve the desired specific surface area and conductivity. Mitlin et al. created three‐dimensional sponge‐like graphene nanoarchitectures via a microwave synthesis process.^139^

The specific capacitance of graphene‐based EDLCs is strongly affected by the surface area for access to electrolyte ions. Chemical activation with potassium hydroxide (KOH) is considered as an efficient method to enhance the surface area for graphene‐based materials.^140^ It is suggested that the activation of carbon with KOH proceeds as the following equation: 6KOH + C = 2K + 3H_2_ + 2K_2_CO_3_. Then the reaction is followed by decomposition of K_2_CO_3_ and/or reaction of K/K_2_CO_3_/CO_2_ with carbon, which generate nanoscale pores during the activation process.

**Figure 6:**
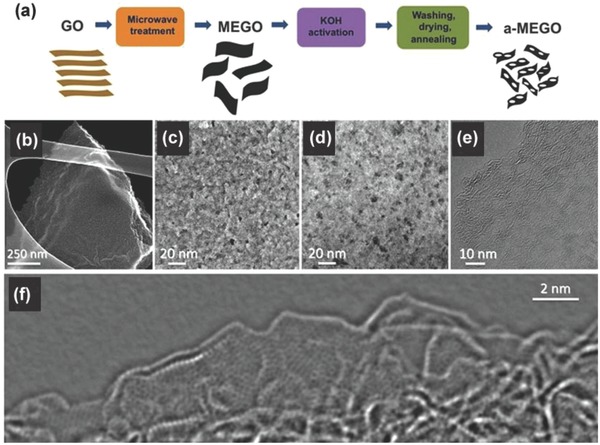
a) Schematic process of the microwave exfoliation/reduction of GO (MEGO) followed by chemical activation with KOH denoted as a‐MEGO. b–d) Low and high magnification SEM images of a‐MEGO. e) High‐resolution phase contrast electron micrograph of the thin edge of a‐MEGO. f) High‐resolution TEM image from the edge of a‐MEGO. Reproduced with permission.^141^ Copyright 2011, AAAS.

Ruoff and coworks have presented their works on activated graphene‐derived materials and their application in SCs.^141^, ^142^ Graphene‐derived carbon with extremely high surface area of up to ≈3,100 m^2^ g^–1^ has been prepared by microwave irradiation and activation with KOH. Microwave irradiation treatment on GO produces a large fraction of micro‐ and mesopores to provide a large and accessible specific surface area for charge accommodation. **Figure** **6**a shows the schematically process of the microwave exfoliation/reduction of graphene followed by chemical activation with KOH. At first, graphene is obtained by reduction of GO by microwave treatment, which is denoted as MEGO. And then, the as‐synthesized MEGO is activated with KOH that generates nanoscale pores while retaining high electrical conductivity. The activated graphene‐based carbon material is denoted as a‐MEGO. The surface area of the activated materials could be controlled by the mass of KOH. During the activation process, graphene is etched and reconstructed into porous networks. Figure 6b–d demonstrate the porous morphology of the activated carbon material with meso‐ and micropores ranging from 1–10 nm. Figure 6e and f illustrate the edge of a‐MEGO with the presence of a dense network of nanoscale pores surrounded by highly curved, predominantly single‐layer carbon and the in‐plane crystallinity. Therefore, a relatively high specific volumetric capacitance and gravimetric energy density are achieved in organic and ionic liquid electrolytes due to improved porosity and enhanced specific surface area. Moreover, this group present a novel method to prepare free‐standing, and flexible porous carbon thin films by chemical activation of reduced GO paper.^143^ After activation, a high specific surface areas of up to 2400 m^2^ g^–1^ and a very high in‐plane electrical conductivity of 5880 S m^–1^ are achieved, which contributes to excellent specific capacitances and energy densities of 120 F g^–1^ and 26 Wh kg^–1^.

Graphene holds its promise for high‐power, flexible electronics because its characteristic feature of two‐dimensional geometry provides high effective surface area for energy storage.^144^ Graphene paper or film are ideal materials for flexible energy storage devices benefiting from their strong mechanic and electrical conductivity. Recent advancement has been achieved on the synthesis and fabrication of flexible graphene films by filtration and assembly of individual graphene sheets.^145^, ^146^ For example, flexible graphene films incorporated with carbon black nanoparticles^147^ and cellulose^148^ are prepared by a simple vacuum filtration method. Carbon black nanoparticles and cellulose are introduced to serve as spacers to mitigate the self‐stacking of individual graphene sheets during the filtration process. Consequently, the resulting flexible electrodes display an improved electrochemical performance compared to the pure graphene paper. However, the further applications of graphene films‐based energy storage devices have been restricted by unsatisfied volumetric performances as a consequence of the compact geometry of the stack and the ion mobility.^149^

**Figure 7:**
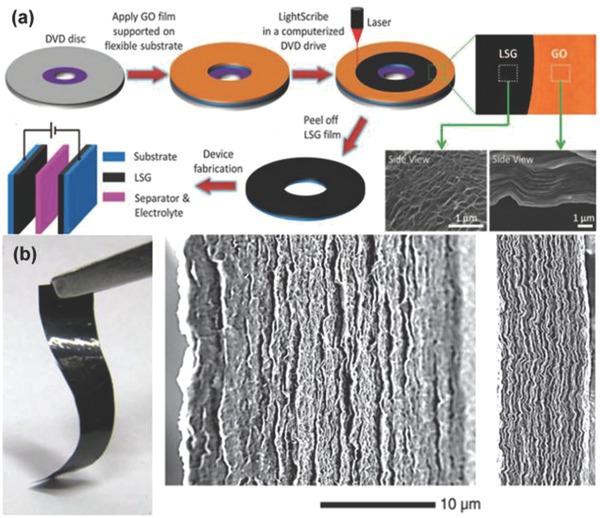
a) The illustration of schematically fabrication process of laser‐scribed graphene‐based SCs. Reproduced with permission.^150^ Copyright 2012, AAAS. b) A photograph showing the flexibility of the resultant liquid electrolyte‐mediated graphene films and SEM images of cross sections of the resultant liquid electrolyte‐mediated graphene films containing 78.9 volume percent (vol. %) and 27.2 vol. % of H_2_SO_4_, respectively. Reproduced with permission.^152^ Copyright 2013, AAAS.

To address this problem, Kaner et al. have developed an all‐solid‐state approach for the production of graphene‐based SCs to avoid the stacking of graphene sheets by a direct laser strategy.^150^ **Figure** **7**a illustrates the fabrication process. At first, a GO film was drop‐cast on a flexible substrate and placed on top of a standard LightScribe‐enabled DVD media disc. Then, an infrared laser was applied inside the LightScribe‐enabled DVD optical drive and reduces the GO film to graphene film. The reduction process can be observed that the film colour changes from golden brown to black and the initially stacked GO were converted into well‐exfoliated laser‐scribed graphene by the analysis of SEM images. The as‐prepared laser‐scribed graphene films possess a high specific surface of 1520 m^2^ g^–1^ and excellent conductivity of 1738 S m^–1^. Moreover, the resultant graphene films can serve as flexible electrodes to be directly assembled into symmetric SCs. The as‐fabricated symmetric SCs exhibit an ultrahigh volumetric energy density up to 1.36 mWh cm^–3^ and a volumetric power density of 20 W cm^–3^ as well as excellent cycling stability.

Additionally, the self‐stacking of graphene sheets could be mitigated by incorporating with carbon or water into internal sheet spaces. Li group have discovered that the resultant wet graphene film after filtration did not occur serious stacking by using water as an effective spacer to prevent the stacking of individual graphene sheets.^151^ The solvated graphene films possess superior specific surface area and conductivity to its corresponding freeze dried films indicating good connection between individual sheets. Consequently, the resultant solvated graphene films could obtain unprecedented electrochemical performances. Subsequently, they acquired porous yet densely packed carbon electrodes by capillary compression of adaptive graphene gel films in the presence of a nonvolatile liquid electrolyte (H_2_SO_4_).^152^ Figure 7b illustrated the flexibility and cross section images of the films containing 78.9 volume percent (vol. %) and 27.2 vol. % of H_2_SO_4_, respectively. The liquid‐mediated dense integration of graphene materials possess high ion‐accessible surface area and low ion transport resistance. In this case, electrochemical capacitors based on the resulting films exhibit a high gravimetric and volumetric capacitances of 200 F g^–1^ and 18 F cm^–3^, respectively as well as the energy densities approach 60 Wh L^–1^.

All these results make it possible to develop new graphene‐based SCs with the combination of high energy density and high power density in terms of gravimetric and volumetric measurements. In addition, transparent flexible thin film based graphene have been synthesized and applied into capacitive touch pad contributed to their high response sensitivity under fast touch rates.^153^

#### 3.1.2 Carbide‐Derived Carbon

Carbide‐derived carbons (CDCs) have received considerable attraction as electrode materials due to their high specific surface area, controllable porosity and high volumetric capacitance. Gogotsi groups have devoted much effort to synthesize carbons derived from metal carbide and investigated their capacitive behaviors.^154^ In general, CDCs are prepared by the selective removal of non‐carbon atoms from the corresponding carbides with halogens in an elevated temperature environment or thermal/hydrothermal decomposition. During the high‐temperature chlorination of metal carbides, a large number of micropores can be produced and the pore size distribution of resultant CDCs can be precisely tuned by changing the chlorination temperature and the carbide precursor.

Various types of carbide precursors have been explored for the fabrication of CDCs such as SiC, TiC, VC and so on. The carbide precursors will lead to the change in terms of pore size distribution. Taking the elevated temperature at 1200 °C as example, CDCs are investigated with a pore size range of 0.8 to 2.1 nm while SiC and B_4_C serve as initial precursors. Elevated temperature is one of key factors for a narrow pore‐size distribution. For example, carbon derived from SiC at 900 and 1200 °C produced an average pore size of 0.65 nm and 1.2 nm. Ti_3_SiC_2_ derived CDCs have been produced at temperatures from 200–1200 °C and precise control of size is achieved an average pore size in the range of 0.6–1.6 nm.^155^ It is an attractive route for the manipulation of the micropore (<2 nm) size distribution. Moreover, the halogenation conditions will affect the resulting pore size and specific surface area and then lead to distinguishing electrochemical performances in either aqueous or non‐aqueous electrolytes. CDCs with mean particle sizes around 20–40 nm have been synthesized by the chlorine treatment of TiC with temperature ranging from 200 to 1200 °C. The CDC produced at 600 °C display the highest capacitance value owing to the high specific surface area and large pore volume.^156^

Since the CDCs can retain the shape of initial carbides, CDCs with different textures and morphologies are capable to be fabricated depending on the morphology of the carbide precursors such as powders, nanopowders, thin films, fibers and so on. For instance, nanofibrous CDC felts have been derived from electrospun TiC nanofelts with nanofiber diameters of 100–200nm.^157^ On account of the conformal transformation of TiC into CDC, the resulting CDC felts conserves the unique properties of precursor including interconnectivity and structural integrity and flexibility. Consequently, CDC nanofelts are developed as flexible electrodes and reveal superior gravimetric capacitance in aqueous and organic electrolytes.

Monolithic CDC films are able to be fabricated on various substrates by dry etching procedure and then the as‐prepared CDC films can be directly used as free‐standing electrodes for EDLCs due to their reduced macropore volume, elimination of polymer binders and good adhesion between current collector and active material. Continuous carbon films have been synthesized by dry etching of sputtered TiC films on glassy carbon, Si and other substrates.^158^ Electrochemical investigation of the resultant CDC film displays a significant increase in the volumetric capacitance up to 180 F cm^–3^ in 1.5 m tetraethyl ammonium tetrafluoroborate (TEABF_4_)/acetonitrile (AN) electrolyte. As the chlorination temperature decreases, the volumetric capacitance held back by the decreasing average pore size which is inadaptable for TEA cation adsorption/desorption in pores.

However, the microporous character of CDC significantly suppress the ion motion within individual particles, which is a drawback for their application in EDLCs requiring the presence of an interconnected hierarchical pore structure to ensure both high capacitances (provided by micro‐ and narrow mesopores) and efficient transport throughout the entire material (provided by larger transport pores). Pore size of CDCs has been tailored by various routes such as polymeric precursor routes, templating approaches, and post‐synthetic activation/annealing procedures.

In particular, hard‐templating approach has been explored to design hierarchical porous CDCs materials. Hierarchical micro‐mesopore CDC mesofoams have been obtained by using mesocellular silica foam as a template in combination with a polycarbosilane precursor.^159^ Ordered mesoporous CDCs have been fabricated using a silica template for the precise regulation of the pore size in the micropore and mesopore ranges.^160^, ^161^ The well‐defined hierarchical pore system serve as ion‐highways and allow for enhanced kinetics in adsorption processes, which would lead to increase in capacitance and response rate. Chemical activation is an attractive approach to develop better electrochemical performances by an increase of surface area and volume of small pores <2 nm. As a consequence, the activated CDC exhibit a high capacitance value of 180 F g^–1^ in organic electrolyte, which is 30% larger than that of untreated samples.^162^

**Figure 8:**
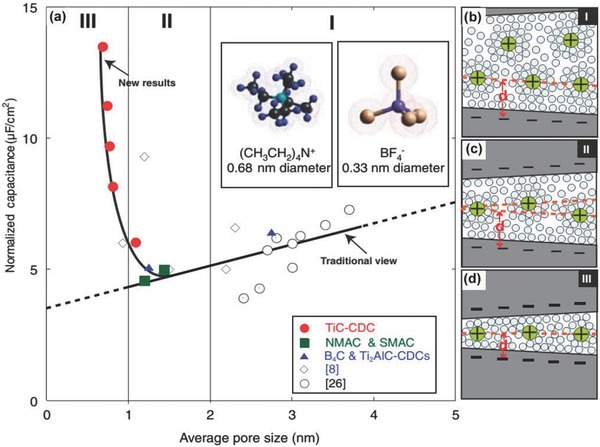
a) Plot of specific capacitance normalized by BET specific surface area and average pore size for the carbons. b–d) Drawings of solvated ions with distance between adjacent pore walls to illustrate distinguishing behavior schematically: (b) greater than 2 nm, (c) between 1 and 2 nm, and (d) less than 1 nm. Reproduced with permission.^163^ Copyright 2006, AAAS.

An anomalous increase has been observed for CDCs‐based SCs while the pore size is less than 1 nanometer.^163^ To explain this behavior, CDCs derived from TiC have been studied in depth on the effect between pore size and normalized capacitance by Brunauer‐Emmett‐Teller (BET) specific surface area. **Figure** **8**a show the plot of normalized specific capacitance and average pore size. It is noticed that a critical pore size value ca. 1 nm is found in this plot. When the pore size below the critical value, the normalized specific capacitance will increase sharply along with decreasing pore size, which is reverse trend with traditional view. Carbons derived from other precursors are also illustrated this phenomenon, indicating that the size effect is independent of the carbon material. Figure 8a is divided into three regions based on average pore size. The diameters of solvated ions are 0.68 and 0.33 nm for (CH_3_CH_2_)_4_N^+^ and BF_4_^–^, respectively. Figure 8b–d illustrates the charge mechanism of solvated ions with various pore sizes. When the pore size is larger than twice the solvated ion size in region I, the capacitance is attributed to the compact layers of ions on both adjacent pore walls. The capacitance is already normalized by the surface area. When the pore size decreased to less than 2 nm but larger than 1 nm, the normalized capacitance is reduced due to reduced surface area for double‐layer charge storage.

However, a sharply increase is found for the normalized capacitance when the pore size approaches the size of the electrolyte ions. Such an anomalous trend in subnanometer pores is ascribed to the desolvation of the electrolyte ions entering subnanometer pores. As the CDC samples were exclusively microporous, the capacitance increase for subnanometre pores clearly shows the role of micropores. Partial or complete removal of their solvation shell was allowing the ions to access the micropores. Hence, the resulting smaller charge‐separation distance between the ion centers and the pore walls leads to greatly increased capacitance. A similar result has been observed by another research group.^164^ More explanation for the anomalous trend have been present by using a heuristic theoretical model and first‐principles density functional theory (DFT) calculations and detailed analyses.^165^, ^166^ Besides, the authors have verified the phenomenon by a recent experiment utilizing an ionic liquid electrolyte with no solvation shell around the electrolyte ions.^167^

#### 3.1.3 Carbon Nanotube

Carbon nanotubes (CNTs) have been widely studied as electrode materials due to the unique open tubular network structure, remarkable mechanical and electrical properties. CNTs can be divided into single‐walled carbon nanotubes (SWNTs) and multi‐walled carbon nanotubes (MWNTs).^168^ Their open‐ended tunnel is beneficial for electrolyte percolation and facilitate the ion diffusion. However, relatively small specific surface area (generally <500 m^2^ g^–1^) limits the energy density of CNTs‐based SCs.^169^

Resilient and robust framework of CNTs makes them a good choice for free‐standing SCs electrode. Flexible SCs using SWCNTs as stretchable electrodes have been studied to enlighten a broad area of stretchable energy‐storage devices. For example, Roy et al. fabricate flexible and substrate‐free papers made from SWCNTs are modified by plasma treatment.^170^ Vertically aligned structures are found on its surface, which benefit the enhanced capabilities of 290 F g^–1^.

CNTs‐based electrodes have been synthesized viachemical vapor deposition method. For instance, Hahm et al. have exploited CNTs and porous carbon nanocups to construct three dimensional hybrid nanostructured electrodes for high‐power and high areal energy EDLCs by using a short channel anodized aluminium oxide as chemical vapor deposition template.^171^ Li et al. have fabricated flexible and deformable electrodes based on CNT sponges with highly porous conductive networks by the CVD method.^172^

Moreover, hybrid flexible electrodes can be readily formed by incorporating the CNTs with foreign species (such as graphene).^173^ Their remarkable mechanical properties make them a good support for active materials. Unique structures for CNTs and graphene are apt to form flexible electrodes without adding extra current collectors, conductive additives or binders.

As a member of carbon family, CNTs inherit intrinsic properties to bridge the defects of graphene sheets and physically separate graphene sheets to prevent aggregation.^174^ A number of approaches have been employed for the fabrication of hybrid graphene/CNTs films including hydrothermal treatment, electrophoretic deposition, CVD, etc.^175^, ^176^ For example, Dai et al. have reported the fabrication of hybrid carbon films by self‐assembly of functionalized two‐dimensional graphene sheets and one‐dimensional CNTs via electrostaticinteractions.^177^ Hybrid SWCNTs/graphene electrodes have been prepared by a simple casting technique for a high energy density SCs device in ionic liquid.^178^ Roy et al. have created three‐dimensional pillared vertically aligned CNT/graphene architectures by rational strategy with tunable length of CNT pillars.^179^

The combination of one‐dimensional CNTs and two‐dimensional graphene sheets display greatly improved electrical, thermal conductivity and mechanical flexibility compared with each of the single components. Significant enhancement has been investigated by combining the advantageous properties of two species, indicating their promising applications for high‐performance SCs.

#### 3.1.4 Porous Material‐Derived Carbon

Porous material‐derived carbon has been regarded as one of the most widely electrode materials owing to their high specific surface area for accumulation of charge.^180^ A porous network can be divided into micropores (<2 nm in size), mesopores (2–50 nm) and macropores (>50 nm) characterized by a broad distribution of pore size. Better‐performing SCs with optimum pore size result from their facilitated ion penetration and accessibility as well as mobility of the ions within the electrode.^181^

**Figure 9:**
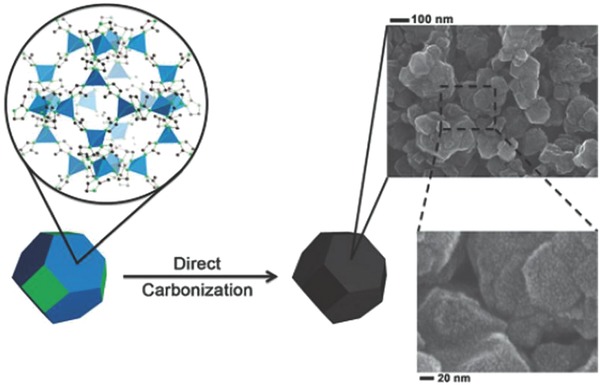
Schematic illustration of preparation of porous carbon via direct carbonization of ZIF‐8 andthe SEM images of the resultant carbon. Reproduced with permission.^186^ Copyright 2012, RSC.

Firstly, metal‐organic frameworks (MOFs) have been investigated extensively as one of porous materials for synthesizing porous carbon with tunable pore structures.^182^ MOFs are typical inorganic‐organic hybrids assembled by transition‐metal clusters and organic molecules using vapor phase or incipient wetness techniques. It is noting that MOFs are thermally decomposable. Therefore, pure porous carbon networks can be produced by direct carbonization without complicated post‐treatment and the pore texture of the resultant porous carbon is determined by the pore characteristics of MOFs.^183^, ^184^ Zeolitic imidazolate framework (such as ZIF‐8) has gained particular attention as sacrificial templates to construct microporous carbons because the zinc metal sublimates or evaporates very easily at high temperature.^185^ Ariga et al. have synthesized nanoporous carbons with high surface area through direct carbonization of ZIF‐8 as illustrated in **Figure** **9**.^186^ Further research has revealed the importance of the pyrolysis temperature for revolution process for porous carbon. The resultant different specific surface area and pore volume is confirmed by nitrogen adsorption measurements. The specific surface areas increased with an increase in the carbonization temperature.

Secondly, various precursors emerge as the most viable materials for the fabrication of mesoporous and macroporous carbons by template techniques or directly carbonization such as natural precursors, biomass, polymer and so on.^187^, ^188^ Traditional carbon sources such as coal, versatile natural and waste materials have been exploited as fantastic precursors to obtain high quality carbon because of their chemical composition and universal resource acquisition.^189^, ^190^, ^191^ However, the complicated process and relatively high cost have hinder their further application. Béguin et al. directly pyrolyze seaweeds under a nitrogen atmosphere to obtain an oxygen‐enriched carbon.^192^ The utilization of waste materials including agricultural wastes, foods, and even animal products decrease not only cost competitiveness but also environmental impacts to meet the urgent need for sustainable development strategies.^193^, ^194^, ^195^ Electrochemical applications are generally influenced by high specific surface area and optimum pore size.^196^, ^197^ Accordingly, the porous structure of carbon can be created after activation with increased specific surface area and pore volume.^198^, ^199^ Activation process make the materials functionally more effective, which is affected by the activation time and temperature.

Thirdly, template carbonization is a particular method for porous carbon with precisely controlled structures and pore size.^200^ In general, hard and/or soft templates are employed to regulate the pore structures by incorporated into the precursors.^201^ And then porous carbon are acquired after the removal of templates. Hard/soft templates and activation approach are reported for the synthesis of hierarchically porous carbon monoliths with tunable porosities. Templates with dimensions at different length scales are exploited to generate the multimodal pores.^202^

Hard template are inorganic oxides such as SiO_2_,^203^ MgO^204^ and so on, and they are usually removed by the use of toxic reagents such as NaOH or HF.^205^ For instance, mesoporous shell carbon nanospheres with high surface area have been synthesized by co‐assembly of monodisperse silica nanospheres method and display a high specific capacitance of 251 F g^–1^ and long cyclic life.^206^ Soft templates are thermally unstable organic polymers thus being removed during the carbonization process. For example, triblock copolymer EO_106_‐PO_70_‐EO_106_ is used as soft template to prepare ordered mesoporous carbonaceous materials.^207^, ^208^ They possess substantially larger pores size of the electrolyte ion leading to solvation shell for high capacitance. Mesoporous channels have a lower ion‐transport resistance and a shorter diffusion route but limited high‐rate capacity.^209^ The application of materials with macro‐porosity is attractive due to fast ions transportation at high rates but limited by relatively low energy density.^210^

**Figure 10:**
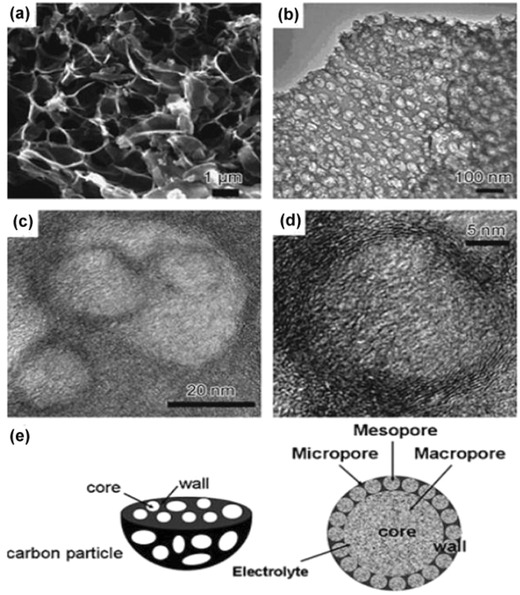
a) SEM image of the macroporous cores, b) TEM image of the mesoporous walls, c) TEM image of the micropores, d) high‐resolution TEM image of the localized graphitic mesopore walls, and e) schematic representation of the three‐dimensional porous texture. Reproduced with permission.^213^

Hierarchical porous electroactive materials are desired for high energy storage attributed to the specially designed pore structures.^211^ Recently, hierarchical porous carbons provide better accessibility and active sites for energy applications by combining well‐defined macropores and interconnected meso‐ and microspores. Such hierarchically porous carbons feature with improved mass transport facilitated by the macropores and high surface area and pore volume from micro‐/mesopores.^212^ Cheng group have developed a three‐dimensional hierarchical porous texture combining macroporous cores, mesoporous walls, and micropores.^213^ **Figure** **10**a displays the texture of the macroporous cores, which serve as ion‐buffering reservoirs. Mesoporous walls and micropores around the mesopores are characterized by TEM images in Figure 10b and c. Mesoporous walls minimize the diffusion distance for electrolyte ion transport and micropores strengthen the charge accommodation. Figure 10d further reveals the existence of localized graphitic structures providing enhanced electric conductivity. Three‐dimensional porous texture is illustrated schematically with the combination of hierarchical porosity in Figure 10e. The unique structure of the resultant carbon is capable to improve energy and power densities in both aqueous and organic electrolytes.

In addition, the recent interest in flexible electronic devices boost textile‐based SCs. Natural cotton composed of cellulose fibers has been used in textiles and clothing. Cotton has been considered as a promising raw material for carbon flexible electrodes due to its low‐cost, light weight and high mechanical flexibility as well as its soft and porous nature.^214^ Carbon flexible materials are obtained through direct carbonized cotton by simple thermal treatment. The resultant carbons possess high surface area, good electrical conductivity and mechanical flexibility. Moreover, the three‐dimensional network of textiles facilitates the access of electrolytes.^215^ These features enable carbon‐derived from cotton as candidates for high‐performance SCs.

#### 3.1.5 Heteroatom‐Doped Carbon

Heteroatom‐doped carbon materials have attracted tremendous attention because of their extensive application in energy conversion and storage devices.^216^, ^217^ Heteroatom doping with foreign atoms is an alternative strategy to tailor the capacitive behaviors of carbon materials through modulating the electronic properties and the surface chemistry.^218^, ^219^, ^220^ Superior capacitive behaviors result from an extra contribution to capacitive enhancement through Faradic reactions due to the heteroatom doping effects. A large number of efforts have been recently devoted to the synthesis of the incorporation of electron‐withdrawing (such as boron) and electro‐donating heteroatoms (such as nitrogen) into carbon frameworks.

Boron (B) acts as electron acceptor and participates in the carbon lattice by substituting for carbon at the trigonal sites.^221^ Even low‐level boron doping have a great effect on the capacitive performance through modifying the electronic structure of carbon electrode. B‐doped graphene‐based materials have been developed using high temperature processes with gas‐phase B sources or autoclave treatments under inert gas as promising supercapacitor electrode materials. However, it is difficult to mass production.^222^ In that case, Han et al. produced more effective B‐reduced GO via a solution process on a large scale.^223^ The borane‐tetrahydrofuran adduct was used as B source and a high specific surface area of 466 m^2^ g^–1^ was achieved with small amounts of Boron components. The supercapacitive performances were tested in two‐ and three‐electrode configurations and demonstrated that energy storage was contributed by ion adsorption on the surface of the materials as well as electrochemical redox reactions. Excellent supercapacitor behavior displayed including high specific capacitance values of 200 F g^–1^, a good rate response and good stability after 4500 cycles with a low resistivity for ion movement.

Nitrogen (N) doping has been widely used to improve the electrochemical capacitance of carbon materials by modifying surface wettability and electronic conductivity and introducing pseudocapacitive behavior.^224^, ^225^, ^226^ N‐doped carbon could be produced by many process with various nitrogen sources, such as ammonia,^227^ hydrazine,^228^ organic amine,^229^ and C_3_N_4_.^230^ Significantly improved specific capacitances and rate capabilities have been demonstrated due to their enhanced electrical conductivity. Up to now, effective approaches have been proposed to introduce N into carbonaceous matrix involving post‐treatment, in situ doping and direct pyrolysis of N‐containing materials.

**Figure 11:**
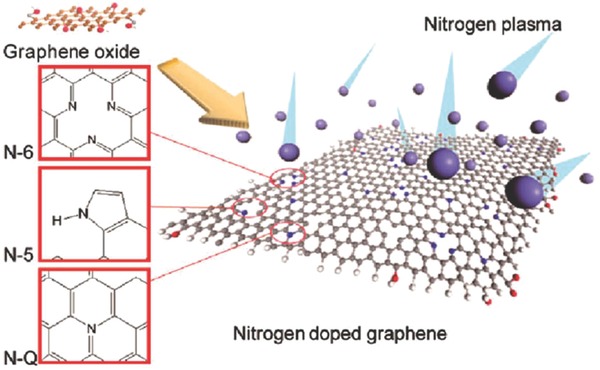
A schematic illustration of the as‐formed nitrogen‐doped graphene by the plasma doping process and possible nitrogen configurations by the doping treatment. N‐Q, N‐5, N‐6 represent graphitic N, pyrrolic N, and pyridinic N, respectively. Reproduced with permission.^231^ Copyright 2011, ACS.

In the post‐treatment procedure, the carbon matrix is generally subjected to ammonia atmosphere at a high temperature. However, it is difficult to control of high‐concentration and uniform nitrogen doping when using ammonia as the nitrogen source. To address these problems, it is of great interest to develop novel nitrogen sources and synthesis methods. Jeong et al. successfully achieved N doping graphene through an effective plasma treatment.^231^ By the plasma process with physical momentum, N atoms replace the existing carbon atoms. Plasma reduction process produces a large number of defect sites which improve the effectiveness of N doping on the graphene basal plane. Further research on microscopic features of N‐configurations, the presence of pyrrolic N, pyridinic N and graphitic N can be noted which are shown in **Figure** **11**. The manipulated local electronic structures allow for enhanced binding with ions in the solution. The electrical conductivity of doped graphene is significantly improved as a result of the restoration of the graphene network by the formation of C—N bonded groups and N‐doping. The improved capacitance of 280 F g^–1^ is obtained due to the N‐configurations at basal planes without sacrificing their excellent cycle life, high power capability, and compatibility with flexible substrates. N‐doped carbon during in situ doping procedure is prepared by incorporating nitrogen‐containing compounds into carbon frameworks followed by carbonization.^232^ For example, Dopamine serve as nitrogen resources to synthesize N‐doped carbons via an evaporation‐induced self‐assembly process.^233^

Relatively speaking, direct carbonization has emerged as a simple procedure to incorporate N into the sp^2^ lattice of carbon. The raw material for pyrolysis can divided into polymers, ionic liquid, nature materials, and biomaterials. By this method, N can be preserved at a relatively large content by adjusting the carbonization temperature.^234^, ^235^ Firstly, porous nitrogen‐containing organic polymers have emerged as versatile substances due to their highly cross‐linked frameworks.^236^ Zhi et al. have developed a novel nitrogen‐rich carbon network derived from terephthalonitrile.^237^ Secondly, ionic liquids have been reported as attractive precursors for the synthesis of heteroatom doped carbonaceous materials via the direct carbonization due to excellent solubility for chemicals and excellent thermal stability features.^238^ Lu groups have exploited ionic liquid functionalized GO as a shape‐directing agent for synthesis of N‐doped microporous carbon sheets.^239^ Thirdly, direct carbonization of sustainable renewable resources has been developed as an efficient strategy to fabricate N‐doped energy storage devices due to the low cost and environmental friendliness. Successful examples have been reported in terms of renewable biological resources provided by nature.^240^ For example, microorganism and protein are available N‐enriched biomass in our daily life. They have been adopted as precursors for large‐scale fabrication of N‐doped materials with high nitrogen concentration.^241^, ^242^

Sulfur (S)‐doped carbon nanomaterials have rarely been investigated for SCs. Chen et al. fabricated S‐doped porous graphene frameworks by directly annealing treatment while dibenzyl disulfide was used as S source.^243^ Parveen et al. have obtained S‐doped graphene via an economical and facile one pot electrochemical methodwhile sodium thiosulphate serve as S source.^244^ S atom are introduced into the framework via covalent bonds and the synergistic effect resulted from sulfur‐doping enhances the electrochemical activity of graphene‐based materials. In addition to mono‐doping, co‐doped materials are also explored for enhance electrochemical performances such as B, N co‐doped,^245^, ^246^ N, S co‐doped,^247^ N, P‐co‐doped,^248^ and N, P, S‐co‐doped materials.^249^ Co‐doped materials have been demonstrated with high specific surface and large pore volume due to synergistic effects from co‐dopants. For graphene, various heteroatoms doping enable that the graphitic carbon atoms in graphene are substituted or covalently bonded by foreign atoms.^250^, ^251^ SCs based on doping graphene utilize the robust charging mechanisms of EDLCs yet exhibit comparable capacitances to those of PCs.

In conclusion, heteroatom in doped carbon materials plays a key role in electron transfer and energy conversion processes. The distinct properties are investigated resulting from various dopants, different doping levels and configuration. The fabrication of heteroatom‐doped carbon with enhanced electrochemical performance provides a viable route to promote its applications in electronic devices.

#### 3.1.6 Carbon Grafting with Surface Groups

It has been witnessed that surface modification by functional group is important for carbon‐based materials in terms of capacitive behaviors.^252^ The surface chemistry of carbon are generally modulated by oxygenic or nitrogenous surface groups to change the hydrophobic/hydrophilic properties such as C=O, —OH, —NH_2_ and so on. The presence of functional groups made the properties of carbon‐based materials improved on three aspects.

Firstly, more facile and effective composite procedures can be developed based on the participation of surface functional groups. Lai et al. have verified that the surface chemistry of graphene is important for the growth of polyaniline.^253^ In addition, the surface characteristics are crucial for electrochemical performances improvement of resultant composites. The amine‐modified composites exhibit the largest increase in capacitance.

Secondly, the surface functional groups enable improved hydrophilicity to allow good accessibility of the electrolyte into the carbon‐based electrode and thus affect the electrochemical properties. A hydrophilic carbon nanotube has been designed by modification with sulfonate groups and applied as electrode materials in on‐chip type solid‐state supercapacitor.^254^ The hydrophilic surface enable good electrolyte affinity and homogeneous dispersibility in water. Consequently, the uniform and dense electrode reveals a high volumetric capacitance and excellent cycling stability due to low ion‐transfer resistance.

Thirdly, specific capacitance and energy density can be enhanced due to the efficient coexistence of EDLCs and PCs derived from surface ion adsorption and the Faradaic reaction of surface functional groups. For example, SWCNTs grafted with carboxylic groups have been developed as electrode materials with a maximum specific capacitance of 146.1 F g^–1^.^255^ Three‐dimensional functionalized multilayer graphene have been developed with controllable surface oxygenic surface groups, which displays high specific capacitance of 508 F g^–1^, highest energy density of 66 Wh kg^–1^ and good stability with 94% retention after 10000 cycles in aqueous electrolyte.^256^

In conclusion, a variety of carbons ranging from zero to three dimensional have been researched extensively as EDLCs electrode, such as graphene, CDCs, CNTs and so on. Carbon‐based materials have been widely explored as electrode materials due to their outstanding electrochemical properties containing excellent cycling stability, fast charging and discharging rate and high power density. However, the energy density of existing carbon‐based SCs is limited, generally an order of magnitude lower than that of batteries.^257^

To address these problems, more efforts have been devoted including carbons with tunable porosity, heteratom‐doping and grafting with surface groups. In the case of porous carbon as electrodes materials, control of structure and morphology play a pivotal role in allowing the effective permeation of the electrolyte to establish electrical double layers for charge storage. Recent development of SCs electrode materials also focus on heteroatoms incorporated carbons. Depending on the type of precursors used in the synthetic step, diverse combination of carbon composition can be prepared. The doped‐species can potentially change the electronic properties of the carbon by altering the electron density on the graphitic surface and affecting the electron affinity of the carbon materials. Recent reports also illustrated that the substitution of carbon with foreign atoms in the graphitic six‐membered ring induces pseudo‐capacitive properties, giving rise to additional enhancement in specific capacitance. Carbon grafting with surface groups have been developed to improve wettability and capacitance performance of carbon materials by chemical surface modification. The charge storage capacity is strongly affected by surface chemistry resulted from the introduction of surface functional groups. The presence of functional species not only increases hydrophilic properties of carbon‐based materials to improve the adsorption and separation properties but also presents pseudo‐capacitive behavior.

### 3.2 Metal Compounds

**Figure 12:**
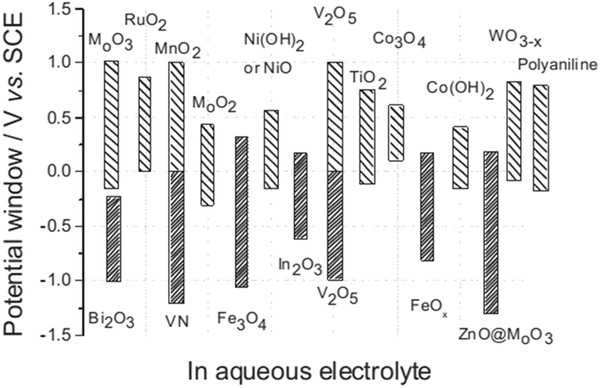
Illustration of the working potential windows of various pseudocapacitive‐type materials in aqueous electrolyte. Reproduced with permission.^259^

At first, transitional metal oxides and graphene‐like layered metal compounds are exciting fields of research resulted from their pseudocapacitive behaviors.^258^ **Figure** **12** summarizes the working potential windows of various pseudocapacitive‐type materials in aqueous electrolyte.^259^ The cathode and anode materials can be distinguished according to the average working potential above or below 0 V (vs. SCE). The cathode materials have been always studied as electrode materials due to their satisfactory capacitive performance. Increased efforts have also been devoted to improve the capacitive performance of anode materials. However, their electrochemical performances have been restricted in terms of cycling stability and power density due to the low electrical conductivity and volume change during cycling process. Various approaches have been attempted for efficient redox charge transfer and improved power density and long‐term stability.

#### 3.2.1 Metal Oxides

Metal oxides have been considered as promising electrode materials for PCs since they can provide a variety of oxidation states for fast surface redox reactions. Ruthenium dioxide and manganese dioxide are two representative redox‐active materials and they have demonstrated satisfied specific capacitance and energy density.

Among metal oxides, ruthenium dioxide (RuO_2_) has attracted much attention as the most promising electrode material for PCs due to their low resistivity, high chemical and thermodynamic stability.^260^ Rutile phase RuO_2_ and hydrated RuO_2_·xH_2_O are two different phase with electro‐active. RuO_2_·xH_2_O has been reported to exhibit a capacitance as high as 1300 Fg^–1^. RuO_2_ represents a high theoretical specific capacitance in wide operational potential (1.4 V) based on their high electronic conductivity and multiple valance states for electron transition (Ru^2+^, Ru^3+^, Ru^4+^, Ru^6+^).^261^ In addition, their highly reversible faradaic response allows them to present good cycling stability. Amorphous RuO_2_ has been investigated and shows higher specific capacitance than the crystalline RuO_2_ due to the flexibility of amorphous state to perform lattice rearrangement upon ion intercalation/deintercalation, allowing more active sites for redox reaction. Typically, amorphous RuO_2_ can exhibit high specific capacitances as high as 1580 Fg^–1^.^262^ Fundamental studies have been done to investigating the electrochemical protonation charge storage mechanism of RuO_2_, which can be simplified as follows: (1) RuO_2_ + xH^+^ + xe^–^ = RuO_2–x_(OH)_x_, 0 < x < 2. (2) RuO_2_ + H^+^ + e^–^ = RuOOH.

Unfortunately, RuO_2_ particles often tend to agglomerations, which significantly degrade their electrochemical performance. Hence, the formation and good dispersion of RuO_2_ particles are of importance to further improve the pseudocapacitive capacitance of the RuO_2_ electrode.^263^, ^264^ In order to utilize both Faradaic and non‐Faradaic processes for large capacity‐charge storage, various RuO_2_/carbon composites with carbon‐based materials were explored as electrode materials for SCs with enhanced energy‐storage capabilities and lower cost.^265^ For example, Naoi et al. have prepared nanosized hydrous RuO_2_/Ketjen Black composites by in situ sol‐gel process induced by ultracentrifugal mechanical force.^266^ The hydrous RuO_2_ nanoparticles are highly dispersed on conductive carbon and exhibit the high specific capacitance of 821 Fg^–1^. Among a variety of carbon materials, CNTs have been employed as supports for composites with RuO_2_ due to their good electron conductivity and accessible surface textures to increasing the utilization of RuO_2_. Hydrated amorphousRuO_2_ nanoparticles are highly dispersed on carboxylated CNTs with the help of bond formation between the RuO_2_ and the surface carboxyl groups of the CNTs.^267^

Graphene features flexible conductive network and rich oxygen‐containing functional groups, which are used to anchor RuO_2_ nanoparticles for enlarged surface area.^268^ The composites benefit from both advantages of RuO_2_ and graphene that offer continuous electron pathways and mechanical integrity. Amorphous hydrous RuO_2_ nanoparticles anchored on the surface of graphene sheets are well dispersed providing a large and accessible surface area as well as effective charge access and propagation. The SCs based on RuO_2_/graphene composites exhibit the high specific capacitance of 570 Fg^–1^, enhanced rate capability, high energy/power density and excellent electrochemical stability of 97.9% retention after 1000 cycles. The electrochemical properties of RuO_2_/graphene composites are superior to pure RuO_2_, which is indicative that the presence of graphene plays an active role in the improvement of electrochemical performance. Besides, more synthesis methods are explored for RuO_2_/graphene composites. For instance, Kim et al. have developed in situ chemical synthesis to prepare RuO_2_/graphene composites.^269^ Choi et al. have reported the integrative assembly of chemically modified graphene building blocks into hierarchical complex structures with the hybrid composition for high performance flexible pseudocapacitors.^270^

Although RuO_2_‐based materials are demonstrated as promising candidates for high‐performance SCs, it is impractical for widespread commercial production due to the high cost of precious metals (Ru) and limited abundance in nature. Therefore, alternative pseudocapacitance materials with low cost have been a major subject in supercapacitor research.

Manganese oxides (MnO_x_) have received tremendous attention for pseudocapacitive electrode materials due to their low cost, natural abundance, and environmental friendliness.^271^, ^272^ For example, Mallouk et al.^273^ and Wang et al.^274^ have reported SCs based on Mn_3_O_4_ with specific capacitances of 121 and 174 Fg^–1^, respectively. Especially, MnO_2_ have been explored as one of promising candidate active electrode materials for SCs with a high theoretical specific capacitance of 1370 Fg^–1^ and large potential window compared to most other metal oxides. MnO_2_‐based pseudocapacitor has been reported for the first time in 1999 by Lee and Goodenough.^275^ MnO_2_ with diverse crystal structures are capable to form different kinds of allotropes resulted from different spatial arrangement of MnO_6_ octahedral.^276^ Besides, MnO_2_ possesses several valence states varying from +2 to +7, which allowing facile electron transition among their oxidation states. Song et al. have reported a mixed‐valent manganese oxide film that displays dramatic performances with anomalous high specific capacitance of 2530 Fg^–1^.^277^ Additionally, MnO_2_ has been a very promising positive electrode for asymmetric SCs.^278^, ^279^

In general, neutral aqueous solution based Li^+^, Na^+^, K^+^ are used as electrolyte for MnO_2_‐based PCs due to their instability in strong acidic and basic media.^280^ MnO_2_ charge storage behavior can be illustrated according to two mechanisms associated with surface and bulk charge storage reactions.^281^ The first mechanism follows a fast faradaic response on the surface adsorption/desorption of electrolyte cations (C^+^) as follows: (MnO_2_)_surface_ + C^+^ + e^–^ ↔ (MnO_2_–C^+^)_surface_. (C^+^ = Li^+^, Na^+^, K^+^.) In this case, the oxidation states of Mn are varied between +4 and +3 resulted from the interfacial reaction while the electrolyte cations maintain charge balance. The second one implies the intercalation and extraction of cations into the crystalline lattice layer of MnO_2_: MnO_2_ + C^+^ + e^–^ ↔ MnO_2_–C^+^. (C^+^ = H^+^, Na^+^, K^+^.) Notably, the electrolyte cations incorporated into the crystalline lattice of layered MnO_2_ and thus the lattice dilation process induces changes in valence state.^282^ However, its further potential applications are primarily hindered by low surface area and intrinsically poor electrical conductivity (10^–5^ to 10^–6^ S/cm).^283^

To enhance the electrochemical performance, a number of approaches have been explored to improve the electrical conductivity and external surface area to achieve full utilization of the active material. Since the electrochemical performances are primarily affected by the surface area, morphology, defect chemistry and pore structures, intensive explorations have been developed as following several aspects: (1) Constructing MnO_2_ nanostructures, (2) incorporating MnO_2_ with carbon matrix, (3) depositing MnO_2_ onto conductive metals, (4) combining MnO_2_ with conducting polymers, (5) coating MnO_2_ onto substrates, (6) designing multiple oxides material.

Firstly, nanostructured MnO_2_ have great potential for further development because the large specific surface area facilitates the improvement of specific capacitance.^284^ The nanostructures of MnO_2_ have been acquired by facile methods and exhibits enhanced electrochemical properties. Roberts et al. have obtained a globular MnO_2_ nanomaterials by a direct precipitation approach.^285^ Jiang et al. have synthesized birnessite‐type ultrathin porous MnO_2_ nanoflowers with narrow pore size distributions.^286^ The electrochemical investigations have revealed an improved specific capacitance.

One‐dimensional nanostructure can introduce fast kinetics and facilitate the electrical transport along the axial direction by reducing the lengths of both electronic transport and ionic diffusion.^287^ One‐dimensional MnO_2_ nanomaterials (nanowires, nanopillars, nanorods, tubular) has been prepared by hydrothermal reactions with high external surface area and exhibits enhanced capacitance and rate capability as well as cycling performance.^288^ For instance, Jiang et al. and Hu et al. have synthesized ultrafine MnO_2_ nanowire networks via a simple process of hydrothermal treatment and followed by subsequent calcinations.^289^, ^290^ Two‐dimensional nanostructures have also been explored for improved pseudocapacitive performance because of high specific surface area and maximal active sites exposed for redox reactions. For example, single‐layer MnO_2_ nanosheet have been prepared by bottom‐up strategy and exhibit a high specific capacitance of 868 F g^–1^.^291^

Secondly, the incorporation of MnO_2_ into a conductive carbon matrix (e.g. graphene, CNT) is an effective way to boost the electrochemical performances of MnO_2_.^292^ The advantages of composites with carbon can be concluded that the presence of carbon enhances not only the electrical conductivity and structural stability but also ease penetration of electrolyte ions.^293^, ^294^ Consequently, the as‐prepared composites provide improved capacitive performances in terms of cycling stability and rate capability. Additionally, the combination of MnO_2_ and carbon guarantee fully utilization of the active surface area and is beneficial for effective electron/charge transfer pathway, which enhance the specific capacitance.^295^

**Figure 13:**
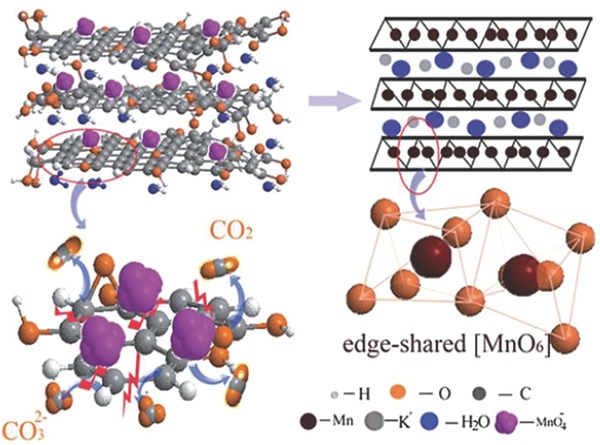
Illustration of the reaction between carbon and potassium permanganate. The upper pictures represent the graphene oxide nanosheets and MnO_2_ nanosheets, and the lower pictures are magnifications. Reproduced with permission.^296^ Copyright 2012, RSC.

Intensive researches have attempted to obtain MnO_2_/carbon nanocomposites using different approaches. Different from other metal oxides and carbon composites, MnO_2_/carbon nanocomposites can be synthesized by the reaction between carbon and potassium permanganate (KMnO_4_) following the equation: 4KMnO_4_ + 3C + H_2_O = 4MnO_2_ + K_2_CO_3_ + 2KHCO_3_. It provides a facile and scalable method for tightly anchoring MnO_2_ onto carbonaceous materials. An intimate contact can be expected between the two hetero‐substances, which not only facilitates electronic conduction but also prevents detachment and aggregation during charging/discharging. Moreover, the electrochemical behaviors could be tailored by adjusting the ratio of carbon and MnO_2_. Hu et al. have synthesized MnO_2_ nanosheets by using GO as a template as shown in **Figure** **13**.^296^ Carbon atoms on the GO framework are replaced in situ by MnO_4_^–^. C—C bonds are broken followed by fully oxidized to CO_2_ or CO_3_^2–^ and the MnO_4_^–^ is reduced to form edge‐shared [MnO_6_] that is unit for MnO_2_. The as‐prepared MnO_2_ nanosheets display a prominent capacitance of 1017 F g^–1^.

Graphene sheets can serve as support and by manganese ions intercalation onto the sheets followed by the nucleation and growth with the help of the chemical interaction between graphene and MnO_2_. For instance, flexible graphene/MnO_2_ paper electrode with large areal mass has been fabricated combining high conductivity of flexible graphene and large specific capacitance of MnO_2_.^297^ In addition, the superior properties of nanostructured MnO_2_/graphene composites make them promising for the use as Faradic electrode in asymmetric SCs. Asymmetric SCs are constructed by MnO_2_/graphene composites as positive electrode and carbonaceous materials as negative electrode.^298^, ^299^ They exhibit high voltage, high energy and power densities in neutral aqueous electrolytes due to the synergistic effects of the two electrodes.

Apart from graphene, other MnO_2_/carbon composites have been employed as electrode materials for SCs applications.^300^, ^301^, ^302^ Yu et al. have assembled MnO_2_ arrays on carbon fiber paper and exhibit a high specific capacitance of 274.1 F g^–1^.^303^ Yu et al. have prepared three‐dimensional bacterial‐cellulose‐derived carbon nanofiber‐network coated MnO_2_ as electrode materials.^304^ The as‐prepared MnO_2_‐based electrodes reveal superior electrochemical properties due to the void volume among coadjacent nanowires. Ozkan et al. have fabricated graphene/MWNT/MnO_2_ nanowire hybrid nanostructured foam via a simple and scalable two‐step process.^305^ CNT and graphene enhance the conductivity and charge transport of the whole electrode. As a result, exceptional capacitive behaviors are demonstrated including a high specific capacitance of 1108.79 F g^–1^, a superior energy density of 391.7 Whkg^–1^, and great capacitance retention over 13,000 cycles. Similarly, Alshareef et al. anchored MnO_2_ nanoflowers onto CNTs and graphene nanosheets as flexible electrode for high‐performance SCs with a specific capacitance of 308 F g^–1^ and a maximum energy density of 43 Whkg^–1^.^306^

**Figure 14:**
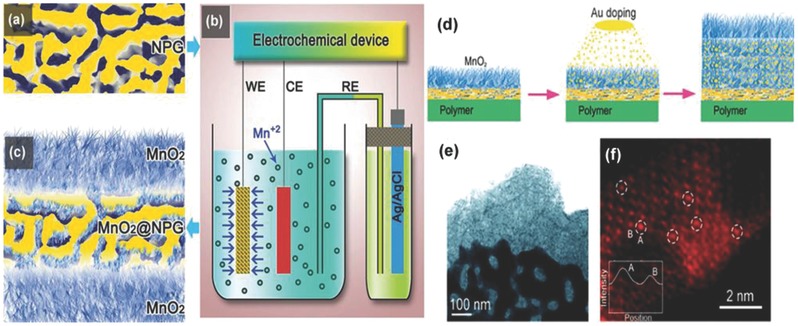
a–c) Schematic illustrations of the fabrication process of MnO_2_/nanoporous gold electrodes by electroplating using a three‐electrode setup (WE: working electrode; CE: counter electrode; RE: reference electrode). Reproduced with permission.^307^ d) Fabrication process of the Au‐doped MnO_2_ electrodes. e,f) STEM images of the Au‐doped MnO_2_. The inset of f) indicates the intensity difference between Au‐occupied columns and undoped Mn‐O atomic columns. Reproduced with permission.^309^

Thirdly, MnO_2_ composites with conductive metals (Au, Ni etc.) have been developed to improve the electrical conductivity and electrochemical utilization while conductive metals serve as current collector and mechanical support. Chen groups have lucubrated the studies of the improved electrochemical properties of MnO_2_ by the addition of nanoporous gold (Au).^307^, ^308^, ^309^ Nanoporous Au is prepared by chemical de‐alloying and acts as a current collector and conductive pathway to provide good electronic/ionic conductivity. MnO_2_ is electroplated onto nanoporous Au to form MnO_2_/Au films as sandwich electrodes for a symmetric architecture as illustrated in **Figure** **14**a–c. The thickness of MnO_2_ films rooting in a nanoporous Au can be adjusted by different electrochemical deposition time and the increase of the MnO_2_ loading amounts brings about gradually decrease of the specific capacitance of the sandwich electrodes. The highest specific capacitance value of 916 F g^–1^ can be achieved. Besides, cycling retention and rate capability is promoted owing to the enhanced electronic and ionic conductivities. Subsequently, the as‐prepared MnO_2_/nanoporous Au films are used as precursors followed by the non‐equilibrium doping of Au via physical vapour deposition as shown in Figure 14d. Figure 14e demonstrates the STEM image of the Au‐doped MnO_2_ film. The high‐angle annular dark‐field STEM image in Figure 14f indicates the homogeneous distribution of the doped Au atoms in the MnO_2_ lattice which are in the form of individual atoms. It is speculated that a better conductivity is achieved since the free Au atoms changes the electronic structure of MnO_2_. To verify the electronic structure of Au‐doped MnO_2_, first principles calculations are performed with two possible atomic configurations Au‐substituted and Au‐interstitial spinel MnO_2_. Consequently, improved capacitive performances are revealed with an ultrahigh specific capacitance of 626 Fg^–1^, and excellent long‐term cycle stability over 15000 cycles.

A number of cost‐effective metals have also been employed as current collectors and/or supports for the growth of MnO_2_. For instance, Xia et al.^310^ have prepared Ti@MnO_2_ arrays and exhibit remarkable electrochemical performance. Self‐supported Ni nanopore arrays has been designed using anodized aluminum oxide as templates.^311^ After depositing MnO_2_, excellent performances are investigated owing to the unique structural features providing large specific surface area and good ion transport. Deng et al.^312^ and Tong et al.^313^ have demonstrated the design and fabrication of Mn@MnO_2_ for high‐performance SCs. The former have obtained three‐dimensionally ordered macroporous Mn@MnO_2_with a satisfactory specific capacitance of 1200 F g^–1^. The later have synthesized MnO_2_/Mn/MnO_2_ sandwich‐like nanotube arrays, which exhibit 937 F g^–1^ and excellent long‐term cycling stability with slight decay over 3000 cycles.

**Figure 15:**
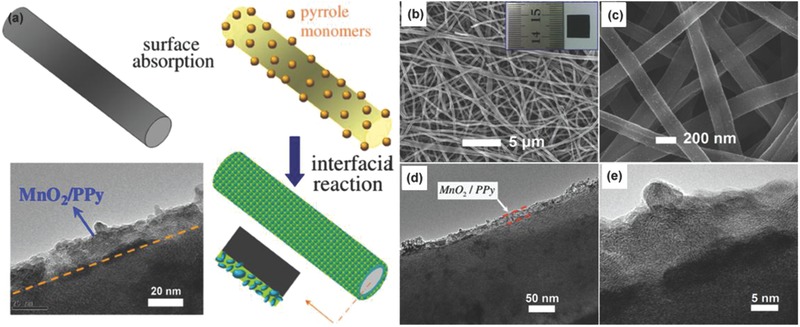
a) Schematically illustration of design and fabrication of MnO_2_/polypyrrole@carbon nanofiber composites. b,c) SEM images of MnO_2_/polypyrrole@carbon nanofiber composites. Inset is the digital photo of the composite paper as freestanding electrode. d,e) TEM and HRTEM images of MnO_2_/polypyrrole@carbon nanofiber composites. Reproduced with permission.^314^ Copyright 2011, RSC.

Fourthly, the introduction of a conducting polymer matrix for MnO_2_ has been a good approach to improve its electrochemical utilization and electrochemical stability. The nanocomposites constructed of MnO_2_ and conducting polymers have been synthesized by previous researches. For example, Kang et al. have presented a flexible nanocables as MnO_2_‐based electrode materials by incorporating MnO_2_ and polypyrrole into carbon nanofiber substratesas shown in **Figure** **15**a.^314^ The triaxial MnO_2_/polypyrrole@carbon nanofibers are synthesized by in situ interfacial redox reaction between permanganate ions and pyrrole according to the equitation in Figure 15b and c. MnO_2_ serves as oxidant for polymerization and polypyrrole coated on MnO_2_ surface to improve their stability as illustrated in Figure 15d and e. The presence of conducting network of polypyrrole provides more access for ion transportation and charge storage process. The free standing electrodes exhibit 705 F g^–1^ and good rate capability as well as long‐term cycling stability due to the synergetic effect among each component.

Fifthly, deposition of MnO_2_ onto highly conductive structures has been considered as a promising strategy. Cui groups have explored intensive researches on SCs fabricated by exploiting paper, textile and sponge as substrates.^315^, ^316^ These substrates with porous structure allow full accessibility of electrolyte to MnO_2_ and then enhance the capacitive performances. The MnO_2_‐CNT sponge exhibits remarkable performance including a specific capacitance of 1230 F g^–1^, ultrafast charge discharge rate and outstanding cycling stability over 10000 cycles.

**Figure 16:**
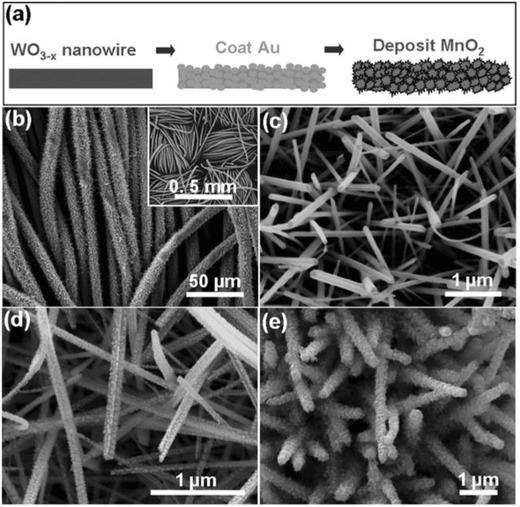
a) Schematic of the fabrication process for WO_3–x_@Au@MnO_2_nanowires. SEM images of the as‐prepared b,c) WO_3–x_nanowires on carbon fabric, d) WO_3–x_@Au nanowires, and e) WO_3–x_@Au@MnO_2_ nanowires on carbon fabric. Reproduced with permission.^319^

Sixthly, efficient MnO_2_‐based electrode materials have been achieved by coating MnO_2_ onto metal oxides constructing core‐shell structures to improve specific capacitance and charge/discharge rate.^317^, ^318^ For instance, Lu et al. have fabricated hybrid WO_3–x_@Au@MnO_2_ core‐shell nanowires on flexible carbon fabric by physical evaporation deposition process as presented in **Figure** **16**a.^319^ As illustrated in Figure 16b and c, WO_3–x_ nanowires are uniformly covered on carbon fabric with the average diameter of nanowires ranging from 40 to 150 nm and length around 5 um. Subsequently, the WO_3–x_ nanowires are coated with gold as shown in Figure 16d and followed by the electrodeposition of MnO_2_ layers in Figure 16e. Outstanding specific capacitance is achieved up to 1195 F g^–1^ companied with excellent long‐term cycling stability. Wang et al. have developed MnO_2_ nanowires@Ni_1–x_Mn_x_O_y_ nanoflakes core‐shell nanostructures for high‐performance with a high specific capacitance of 657 F g^–1^, and stable cycling stability.^320^ The core metal oxides with good electronic conductivity generally benefits the enhancement for MnO_2_‐based electrodes due to larger contact area with the electrolyte ions and electron transfer. Hierarchical nanostructures exhibit superior electrochemical properties due to the synergic effects among different components.

Nickel oxide (NiO) has been widely investigated as a promising candidate for energy storage applications due to its low cost, environment‐friendly, high chemical and thermal stability. When NiO‐based materials are investigated by CV curves, a pair of cathodic and anodic peaks is revealed due to the reverse redox process between NiO and NiOOH as following: NiO + OH^–^ = NiOOH + e^–^.^321^ Intensive efforts have been devoted to develop facile and effective synthesis of NiO nanostructures. Firstly, NiO can be obtained by annealing treatment from Ni.^322^, ^323^ Secondly, NiO is obtained by the thermal annealing treatment of Ni(OH)_2_ prepared by microwave, hydrothermal and electrodeposition method, etc.^324^, ^325^

The pseudocapacitive behavior of NiO depends on the morphology of electroactive materials. One dimensional porous structure has been considered one of most efficient morphologies for enhancing the pseudocapacitance.^326^ For example, Lou group have explored one‐dimensional NiO belts for high‐performance SCs with a high pseudocapacitance of 600 F g^–1^ and remarkable cycling stability over 2000 charge/discharge cycles.^327^ NiO sheets^328^ or nanoflakes^329^ are anchored on substrates through a facile directed method to acquire one‐dimensional hierarchical hollow nanostructures with the advantages of short ion and electron transport pathways. Two‐dimensional NiO sheets are capable to accommodate the structural alteration. NiO sheets have been exploited for high performance pseudo‐capacitance materials.^330^, ^331^, ^332^, ^333^

Despite the great progress, the capacitance is still far below satisfactory value. High resistivity of NiO and large volume change during the cycling processes hinder the further development. Therefore, most studies have been developed aimed at improving the pseudocapacitive performances by fabricating NiO combining with carbon materials, and integrating with other metal oxides. The superiority of NiO/carbon composite could be concluded that the inclusion of carbon materials can improve the electrical conductivity, the dispersibility of NiO and the structural integrity.^334^ Lee et al.^335^ and Lin et al.^336^ have synthesized graphene/NiO and CNTs/NiO composites as electrode materials for high‐performance SCs. Nitrogen‐rich carbon hollow spheres are also developed encapsulate NiO and perform a highest specific capacitance of 1130 F g^–1^.^337^ NiO/metal oxides composites have been explored as active pseudocapacitor material, wherein the metal oxides with high electrical conductivity(eg. TiO_2_) have been introduced to prepare binary composites for enhanced electrochemical performance. For example, Huang et al. have reported NiO‐TiO_2_ nanotube array electrode using a facile one‐step anodization method.^338^ When evaluated as electrode materials, the hybrid nanostructures exhibit an advanced performance.

Co‐based oxides (Co_3_O_4_, CoO) have long been considered as very promising electrode materials by means of their excellent pseudocapacitive properties, such as good redox activity and reversibility.^339^ Methods of preparation of cobalt oxides has been widely used including the sol‐gel process, physical vapor deposition, chemical precipitation and electrochemical deposition.^340^ Control of morphologies and microstructures by the synthesis methodology has a profound effect of the supercapacitive performance of the material due to dissimilarities in the electrode/electrolyte interface properties and ion transfer rates during the charge storage processes.^341^ However, the observed specific capacitances of actual cobalt oxides pseudo‐capacitors are far less than the theoretical prediction especially at high rates due to their poor electrical conductivity and limited surface area.

To overcome these drawbacks, hierarchical porous morphological structures have been regarded as one of most efficient approaches by allowing fast ion/electron transfer and alleviating the structure degradation caused by volume expansion during the cycling process. Bacterial and polystyrene are used as templates for the synthesis of porous Co_3_O_4_ structures.^342^ Metal‐organic frameworks have been used as precursor to obtain porous hollow Co_3_O_4_ with rhombic dodecahedral structures, which exhibit a high specific capacitance of 1100 F g^–1^.^343^ The as‐prepared porous Co_3_O_4_‐based electrodes perform excellent cycling stability due to its hierarchical porous morphological characteristics.

Additionally, incorporation with highly conductive nanostructured carbons (e.g., carbon nanotube, carbon fibers, or graphene) are explored to enhance their supercapacitor performances.^344^ Co_3_O_4_ are directly contact with the substrate without the tedious process of mixing active materials with binders and thus all nanostructures participate in the electrochemical reaction. Moreover, the decreased internal resistance of the electrode facilitates the diffusion of electrolyte into the inner region of the electrode.^345^, ^346^, ^347^

Moreover, a new generation of composites based on Co_3_O_4_ and graphene have being intensively engineered for enhanced performances.^348^ The addition of graphene not only reduce agglomeration but also offer an enhanced surface area to improve their capacitive performances. For instances, Co_3_O_4_ on three‐dimensional graphene foam have been synthesized as a free‐standing electrode with a high specific capacitance of 1100 F g^–1^ and excellent cycling stability.^349^ Well‐designed flexible Co_3_O_4_/rGO/CNTs hybrid paper deliver high specific capacitance of 378 F g^–1^ with excellent electrochemical stability.^350^

To overcome the drawback of low electronic conductivity, Co_3_O_4_ are often directly deposited on metal foils by a large number of methods, such as coprecipitation and template methods or hydrothermal based technology and so on.^351^ Ni foam is chosen as the substrate due to its high electrical conductivity and a desirable three‐dimensional porous structure which can reduce ionic and electronic diffusion distance and provide large electrode/electrolyte contact area.^352^, ^353^, ^354^ The growth of Co_3_O_4_ on Ni foam endows fast ion and electron transport, large electroactive surface area, and excellent structural stability. Superior pseudocapacitive performances are achieved with ultrahigh specific capacitance and excellent cycling stability.

Conducting polymers (such as polypyrrole) are generally immobilized onto the surface on cobalt oxides as a nanosized thin layer to enhance the conductivity and electrochemical stability of the cobalt oxides electrode. Polypyrrole with highly electrical conductivity is anchored to the surface of CoO, facilitating the electron transport and the electrical connection with the current collector.^355^ Further application for the hybrid as an aqueous asymmetric supercapacitor device is also investigated with high energy density of 43.5 Wh kg^–1^ at a maximum voltage of 1.8 V.

Another desirable strategy is exploited by engineered nanostructures with other metal oxides. Particularly, their mixed‐oxide forms exhibit far better electrochemical performance than either monometallic oxide due to the enhanced electrochemical reversibility and conductivity.^356^ Mixed oxides based on cobalt and nickel such as CoO nanowire@nickel hydroxidenitrate is coated on nickel foam with robust hierarchical porosity and high specific surface area by a facile two‐step hydrothermal route. The hybrid structure has combined the merits of the two active components and electrochemical characterization shows a specific capacitance of 798.3 F g^–1^, good rate performance and excellent electrochemical stability.^357^ Co_3_O_4_/NiO core/shell nanostructure arrays on nickel foam are fabricated and characterized as supercapacitor electrodes. A high specific capacitance of 853 F g^–1^ and an excellent cycling stability are achieved owing to the unique porous core/shell nanowire array architecture, and a rational combination of two electrochemically active materials.^358^ Mixed oxides based on cobalt and manganese have also been fabricated by many methods. For example, Kong et al. has fabricated a highly ordered three‐dimensional Co_3_O_4_@MnO_2_ hierarchical porous array on nickel foam by a facile, stepwise hydrothermal approach.^359^ Liu et al. has developed a new and cost‐effective strategy to produce hierarchical hybrid nanostructure arrays based on Co_3_O_4_@MnO_2_ core/shell for supercapacitor applications using three‐dimensional ordered amorphous carbon layers as a sacrificial reactive template.^360^ Xia et al. fabricated a new type of integrated electrodes based on Co_3_O_4_ and MnO_2_ with an optimal design of structure and thus outstanding performances are revealed due to a synergistic effect.^361^ The electrochemical performance relies not only on the exploitation of active materials, but also on the rational design of superior electrode architectures.

Additionally, other oxides have also exploited as pseudocapacitive materials such as vanadium oxides, iron oxides and so on. Vanadium oxides (V_2_O_5_) exhibits numerous oxidation states (V^5+^, V^4+^, V^3+^, V^2+^) similar to that of ruthenium, which renders the oxide unsuitable for use in high‐rate electrochemical devices. Besides, the unique layered crystalline structure of V_2_O_5_ renders them with redox pseudocapacitance on the surface.^362^

Past decades have witnessed that V_2_O_5_ is a promising anode electrode materials. However, its poor electronic conductivity and high dissolution in liquid electrolyte are detrimental to high‐rate and long‐term cycling performance in electrochemical devices. The combination of V_2_O_5_ with CNTs has been demonstrated to be an effective strategy to improve electronic transport but this kind of composite cannot prevent vanadium dissolution.^363^ The addition of CNTs enhances the electrical conductivity of the composites. As a result, CNTs‐V_2_O_5_ composites electrode exhibits excellent rate capability, high capacity, and cycling stability.

Nonetheless, the high dissolution in liquid electrolyte is still problematic. To address this problem, electronic conductive polymers (polypyrrole, polyaniline) are incorporated with V_2_O_5_ for preventing vanadium dissolution. Inspired by this concept, Qu et al. obtain core‐shell structured polypyrrole@V_2_O_5_ nanocomposites, where polypyrrole is grown uniformly on the surface of V_2_O_5_ nanoribbon.^364^ The combination of V_2_O_5_ nanoribbons and polypyrrole is expected as promising electrode material for SCs. On one hand, the morphology of nanoribbons leads to improved charge transfer. On the other hand, polymeric coating of polypyrrole not only enhances the electronic conductivity but also keeps the electrode stable in electrolyte.

Among various traditional metal oxides, iron oxides have emerged as promising candidates due to its low cost, natural abundant resources and environmentally benign nature, such as magnetite (Fe_3_O_4_) and hematite (Fe_2_O_3_).^365^ The charge storage mechanism of iron oxides involves the reversible transition of Fe^3+^/Fe^2+^ redox couple. However, their intrinsic poor electrical conductivity and limited surface area restricted their application in high‐performance SCs. To address this problem, composite materials with other oxides have been developed for enhanced electrochemical performances. Yang et al. design three‐dimensional thin films constructed of Ta_2_O_5_ nanotubes and carbon‐coated Fe_2_O_3_ nanoparticles.^366^ Ta_2_O_5_ nanotubes are selected as active support materials to uniformly anchor carbon‐coated Fe_2_O_3_ nanoparticles. In addition, thin films with high surface area allow for more exposed electroactive sites.

Another promising methods is the direct fabrication of hybrid nanostructured electrodes by integrating iron oxides with a carbon host, where the carbon host serves as the conductive network.^367^, ^368^ On one hand, one‐dimensional carbon nanofibers and CNTs have been exploited as substrates to disperse iron oxides to enhance the electrical conductivity. The incorporation of CNTs leads to a decrease of internal resistance and an improvement in the ion diffusion behavior of the Fe_2_O_3_ films electrode. On the other hand, previous research has demonstrated that modifying hematite with graphene is an effective strategy to improve its electrochemical properties.^369^ The high surface area and high conductivity of graphene allow for effective ion and charge transport.

It is worth noting that iron oxides are capable to show pseudocapacitance at low potential range (below 0 V vs. SCE). Hence, iron oxides have been expected to replace the conventional carbon‐based anodes in asymmetric SC.^370^ For example, asymmetric SCs have been constructed using MnO_2_‐based materials as cathode electrode and Fe_2_O_3_‐based materials as anode electrode.^371^ The as‐fabricated asymmetric SC can operate within the window potential of 2 V and display a high energy density of 50.7 Wh kg^–1^ at a power density of 100 W kg^–1^ as well as excellent cycling stability. Wang group have constructed an asymmetric solid‐state SCs using MnO_2_ as the positive electrode and Fe_2_O_3_ as the negative electrode.^372^ Carbon cloth are used as substrates for the growth of metal oxides as electrode materials. Positive electrode materials are obtained by the growth of MnO_2_ nanowires on carbon cloth via a facile scalable wet chemical method and Fe_2_O_3_ nanotubes are used as negative electrode materials which are prepared by using ZnO nanowires as templates. The device is expected to exhibit enhance specific capacitance and energy density due to the pseudocapacitance in both electrodes. Excellent performances have been achieved including a large potential window of 1.6 V, high rate capability and a high energy density of 0.55 mWh cm^–3^.

#### 3.2.2 Graphene‐Like Layered Metal Compounds

Graphene‐like layered metal compounds have been developed as electrode materials such as metal nitride, dichalcogenide/selenide and carbide.^373^ These layered metal compounds are two‐dimensional nanosheets with atomic thickness and unique physicochemical properties. Recent progress has demonstrated that two‐dimensional metal compounds are of particular interest for high‐performance solid‐state SCs. The superior electrochemical properties benefit from their high specific surface area and extra pseudocapacitive contribution of metal centers.^374^, ^375^

Firstly, metal nitride such as vanadium nitride (VN) has been exploited as a new class of electrode materials for high‐performance SCs due to the good electronic conductivity combined with the variety of oxidation states. Kumta et al. have demonstrated the possibility of VN as electrode material for the first time.^376^ The nanometer‐sized crystals and the high surface area of the nitrides provide the increased susceptibility for surface oxidation and more redox‐reaction sites.

**Figure 17:**
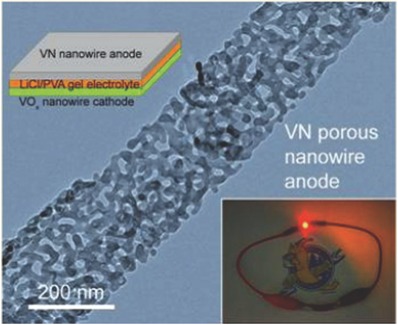
Schematic illustration of quasi‐solid‐state asymmetric device based on porous VN nanowire anode and VO_x_ nanowire cathode. Reproduced with permission.^377^ Copyright 2013, ACS.

VN holds great promise as anode material for asymmetric SCs due to its large specific capacitance, high electrical conductivity, and wide operation windows in negative potential. For instance, Lu et al. have demonstrated a high energy density, stable, quasi‐solid‐state asymmetric device based on porous VN nanowire anode and vanadium oxide (VO_x_) nanowire cathode for the first time.^377^ Herein, porous VN nanowire arrays grow on carbon cloth substrate to avoid the use of binder as shown in **Figure** **17**. The good electrochemical performances of VN‐based electrodes have been achieved including an ultrahigh specific capacitance of 1340 F g^–1^ in 1 m KOH electrolyte. Then the VN is stabilized by using LiCl/poly(vinyl alcohol) (PVA) gel electrolyte to prevent their reversible electrochemical oxidation reaction of forming (VO_x_). Consequently, the electrochemical stability is improved by suppressing the oxidation reaction and structural pulverization, which paves a new ways in design and fabrication of high‐performance VN‐based devices. The asymmetric device exhibits a stable electrochemical window of 1.8 V and a remarkable volumetric energy density of 0.61 mWh cm^–3^ and a high power density of 0.85 W cm^–3^.

Lightweight, thin, and flexible freestanding VN nanowires/CNTs hybrid electrodes have been fabricated by simply vacuum‐filtering method.^378^ VN nanowires are synthesized through hydrothermal synthesis and subsequent thermal nitridation under NH_3_ atmosphere. Solid‐state flexible SCs based on VN nanowires/CNTs with H_3_PO_4_/PVA are demonstrated high performances including a high volume capacitance of 7.9 F cm^–3^ and energy and power density of 0.54 mWh cm^–3^ and 0.4 W cm^–3^ for the whole cell.

Secondly, nanostructured transition metal chalcogenides are promising candidates for efficient solid‐state SCs due to their unique electronic structures and physical properties such as conductivity, mechanical and thermal stability.^379^, ^380^ A large library of metal‐based (including V, Co, Mo, Se, Ge and so on) chalcogenides materials have been exploited for high‐performance solid‐state SCs owing to their special geometric structures with weak interlayer Van de Waals coupling, variable composition, and rich phase structure. Vanadium disulphide (VS_2_) and molybdenum disulphide (MoS_2_) are typical family members of transition‐metal dichalcogenides. Analogous to graphene, they are layered compounds with synergic properties of metallic nature and exfoliative characteristic brought by the conducting S–V–S layers stacked up by weak van der Waals interlayer interactions.

The unique and complicated two‐dimensional electron correlations among V atoms enable VS_2_ great potential as high‐performance in‐plane supercapacitor electrodes due to the planar metallic nature of VS_2_ nanosheets. Feng et al. have developed an all‐in‐solution route to synthesize VS_2_ phase using ammonia as an intercalation to exfoliate bulk VS_2_ flakes into ultrathin VS_2_ nanosheets.^381^ Ultrathin VS_2_ nanosheets are used to assembly highly‐oriented VS_2_ thin films with synergic advantages of high conductivity and high specific area by vacuum filtration. Capacitive properties of as‐obtained VS_2_ films are characterized in 150 nm in‐plane configuration. Electrochemical results reveal a considerable specific capacitance of 4760 µF cm^–2^, and an excellent cycling behavior with no obvious degradation even after 1000 charge/discharge cycles. VS_2_ have been used as anode materials for the asymmetric electrochemical capacitors and delivers a remarkable energy density of 7.4 Wh kg^–1^ (based on the weight of entire device) at the average power density of 3000 W kg^–1^.^382^

**Figure 18:**
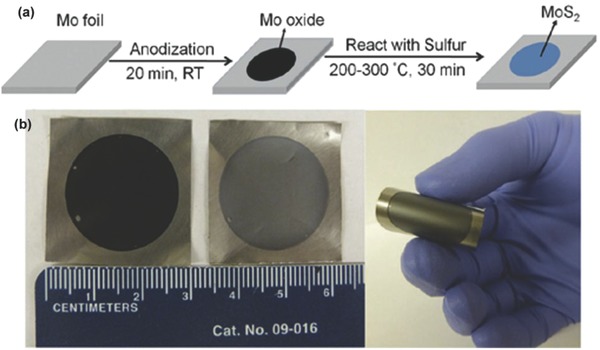
Schematic of the fabrication process and photographs of the flexible electrodes. a) Schematic of the fabrication process and b) photographs of the flexible electrodes. The left photograph shows the visible difference between the Mo oxide (dark color) and MoS_2_ (gray color). The right photograph shows the flexibility of the edge‐oriented MoS_2_ film. Reproduced with permission.^384^

MoS_2_ has attracted much attention due to their typical pseudo‐capacitance behavior resulted from variable oxidation states of Mo atoms ranging from 2 to 6.^383^ Edge‐oriented MoS_2_ thin films have been fabricated by a simple and scalable method as illustrated in **Figure** **18**.^384^ At first, molybdenum metal is electrochemical anodized forming nanostructured Mo oxide porous thin‐films. And then, edge‐oriented MoS_2_ thin films are fabricated after the reaction between Mo oxide thin‐films with sulfur vapor. The as‐prepared MoS_2_ thin‐films are used as electrode materials for solid‐state SCs based on their excellent flexibility. Excellent performances are revealed with an areal capacitance up to 12.5 mF cm^–2^ at 50 mV s^–1^ derived from the high surface area nanoporous morphology.

Higher specific capacitance and better cyclic stability can be achieved by incorporation with pseudocapacitive materials. For example, hierarchical nanostructured MoS_2_@Ni(OH)_2_ nanocomposites have been synthesized by a facile single‐mode microwave hydrothermal technique.^385^ Solid‐stated SCs based on MoS_2_@Ni(OH)_2_ exhibit superior performances than those of MoS_2_ and Ni(OH)_2_ due to the synergistic effect. Multifunctional MoS_2_@ polyaniline have been constructed by MoS_2_ thin nanosheets and PANI nanoarrays via a large‐scale approach and investigated as pseudocapacitor electrodes with a high energy density of 106 Wh kg^–1^.^386^ The crystalline Ni_3_S_4_@MoS_2_ have been designed with hierarchical nanostructures by a facile one‐pot process.^387^ The as‐obtained Ni_3_S_4_@MoS_2_ electrode display a high specific capacitance of 1440.9 F g^−1^ at 2 A g^−1^, which is 1.6 times as that of the counterpart.

Thirdly, metal selenide have been developed as electrode materials in flexible, all‐solid‐state SCs due to their unique electronic. Zhang et al. have developed the phase‐controlled synthesis of two‐dimensional tin selenide (SnSe and SnSe_2_) nanostructures.^388^ High areal capacitances, good cycling stabilities, excellent flexibilities, and desirable mechanical stabilities are observed for both SnSe and SnSe_2_ devices showing their promising applications as a flexible power supply. Wang et al. have synthesized three‐dimensional GeSe_2_ nanostructures by using a CVD method and the as‐prepared materials are applied as electrode materials for flexible all‐solid‐state SCs.^389^ An impressive capacitive behavior are demonstrated arising from their combination of Faradic and nonFaradic processes including a high specific capacitance of 300 F g^–1^, outstanding rate capability and long cycling performances.

Fourthly, transition metal carbides for solid‐state SCs have been explored in past years.^390^, ^391^, ^392^ The low cost and good chemical resistance of the transition metal carbides render them excellent candidates for the next generation of SCs. MXenes, a novel family of two‐dimensional metal carbides have proved to be promising candidates for SCs with volumetric capacitance exceeding most previously reported materials.^393^

Gogotsi group have devoted their research on one of MXenes Ti_3_C_2_, which is both metallically conducting and hydrophilic. At first, Ti_3_C_2_ can be produced by the selective etching from titanium aluminium carbide (Ti_3_AlC_2_) in concentrated hydrofluoric acid (HF).^394^ As predicted theoretical calculations, Li^+^ ions should diffuse rapidly on Ti_3_C_2_ surfaces and result in high storage capacities. Inspired by this predication, they intercalate Li^+^, Na^+^, Mg^2+^, K^+^, NH_4_^+^, and Al_3_^+^ ions between the two‐dimensional Ti_3_C_2_T_x_ layers to exfoliate multilayer Ti_3_C_2_T_x_ form few layers of Ti_3_C_2_T_x_ paper as flexible electrodes.^395^ To some extent, electrochemical performances have been sharply enhanced in aqueous electrolytes. The highest volumetric capacitance has been yielded up to 350 F cm^–3^.

**Figure 19:**
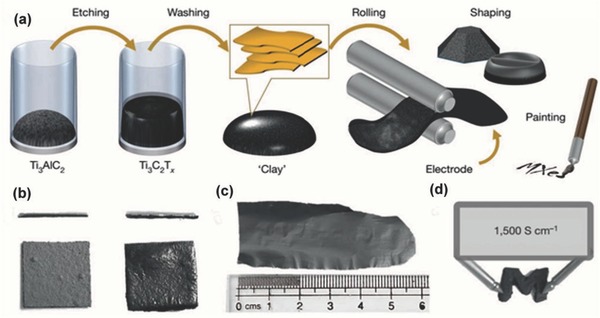
a) The etching process from Ti_3_AlC_2_ to Ti_3_C_2_ clay. b) The cross‐section and top view of the dried samples (left) and hydrated samples (right). c) Image of a rolled film. d) ‘Clay’ shaped into the letter M. Reproduced with permission.^396^ Copyright 2014, Nature Publishing Goup.

However, concentrated HF is hazardous and requires careful post treatment. Subsequently, they have reported a method to synthesis Ti_3_C_2_ by using a solution of lithium fluoride (LiF) and hydrochloric acid (HCl) as etching agents.^396^ As shown in **Figure** **19**a, Ti_3_AlC_2_ is immersed in the solution containing (LiF) and (HCl) followed by a heat treatment at 40 °C for 45 h. The etched material is referred to as Ti_3_C_2_T_x_, where the T denotes surface terminations, such as OH, O and F. As a result, clay like paste of MXenes is obtained after washing with water to remove reaction products and raise the pH towards neutral.

In particular, the wet MXenes could be rolled and shaped to be flexible films with tens of micrometres thick. The as‐prepared wet films will shrink after drying and the dried samples can be rehydrated with swelled volume as shown in Figure 19b. Figure 19c reveals the rolled film with a length of several centimeters and Figure 19d shows that MXenes clay is shaped into the letter M with a high conductivity of 1,500 S cm^–1^ after drying. Electrochemical investigation of the as‐prepared titanium carbide ‘clay’ films display an excellent performances including volumetric capacitances up to 900 F cm^–3^, with excellent cycleability and rate performances. To further improve the electrochemical performance of MXenes, a simple, scalable, and effective hierarchical structure is required to delaminate the stacking of MXene flakes for enhanced electrolyte ions transport.^397^ For example, CNTs are introduced as interlayer spacers and then MXene/CNTs composite is assemble into sandwich‐like flexible papers. Compared to pure MXene, the as‐synthesized composite electrodes exhibit enhanced performances.

### 3.3 Conducting Polymers

Conducting polymers and their derivatives have been extensively investigated as promising materials for high‐performance SCs, such as polyaniline (PANI), polypyrrole (PPy), 3,4‐ethyelenedioxythiophene (PEDOT). Especially, PANI and PPy have been most extensively used as pseudocapacitive electrode materials owing to their high electrical conductivity, environmental stability, excellent capacity for energy storage and relative ease of synthesis.

PANI attracts much attention due to high theoretical specific pseudocapacitance (theoretically 2,000 F g^–1^ in sulfuric acid) and unique fast redox and acid‐base doping/dedoping properties.^398^ PANI exhibits satisfied electrochemical properties not only by virtue of its high surface area and porous nature, but also the presence of two interconvertible benzenoid and quinoid rings by electron transfer associated with its variable oxidation state.

PPy has been explored as one of the most promising polymers for PCs because the monomer (pyrrole) can be easily oxidized and PPy is water soluble. PPy is typically doped with single‐charged anions such as Cl^–^, ClO_4_^–^ and SO_3_^–^, while physical crosslinking of the polymer would occur if doped with multiple‐charged anions, e.g. SO_4_^2–^.^399^

Conducting polymers exhibit both the electrical double layer derived from the surface electrostatic reaction and pseudocapacitive charge storage mechanisms resulted from the facile electron transition along the polymeric π‐conjugated chains. Conductive polymers can be divided into p‐doped with anions in oxidation reaction or n‐doped with cations in reduction reaction expressed as following: (1) Undoped polymer + nA^–^ = polymer^n+^(A^–^)_n_ + ne^–^. (2) Undoped polymer + nC^+^ + ne^–^ = polymer^n–^(C^+^)_n_.

The conducting polymers are usually electrochemically or chemically synthesized through the polymerization of their corresponding monomers.^400^ Chemical polymerization is a universal affordable technique and easy for fabrication for large‐scale devices. Their electrochemical properties have strong correlation with their crystal structures, hydration properties and surface area. Good intrinsic conductivity change from a few S cm^–1^ to 500 S cm^–1^ in the doped state.

However, their application in high‐performance devices is limited due to the poor stabilities during the charge/discharge process as a result of ion doping and dedoping. Large volumetric swelling and shrinking often leads to structural breakdown and thus fast capacitance decay and limited cycling life. A number of approaches have been demonstrated to overcome the mechanical degradation problem to improve the cycling stability of polymer‐based PCs by using binary or multiple composites based on conducting polymer and other electro‐active materials.

Compared to pure conducting polymers, the binary or multiple composites are much more competitive to attain improved performance for supercapacitor application. Binary or multiple composites are obtained by the incorporation of conducting polymers and other electro‐active materials such as metal oxides, EDLC‐based materials.^401^ For example, ternary pseudocapacitor based on PANI, PPy and carbon has been fabricated with excellent cycling stability.^402^ The cycling stability of PANI and PPy electrodes is enhanced by deposition of a thin carbonaceous shell onto their surface. The presence of carbonaceous shell prevents the structure of polymers from breakdown and thus the polymer electrodes exhibit long‐term cycling stability with 85% capacitance retention after 10,000 cycles.

Combined with carbon materials (graphene and CNTs) have open up opportunities for fabricating stable and flexible conducting polymer electrode materials with good stabilities for pseudocapacitive devices.^403^ Carbon materials and conducting polymers can be incorporated through vacuum filtration,^404^ in situ polymerization,^405^, ^406^ and Layer‐by‐layer (LbL).^407^, ^408^ Hybrid materials after optimum are expected to display high capacitances and improved stability due to the synergic effect of the two components.

Compositing conducting polymers with graphene is promising to reduce the “dead volume” during charging and discharging process. Graphene with high specific area can serve as a stable and underlying conductive network to host the active polymer. Moreover, hybrid materials based on graphene and conducting polymers have triggered interest since graphene is a good electron acceptor and conducting polymer is a very good electron donor. Superior rate performance is revealed due to their facilitated ion transport. The composite is covalently functionalized that ensure the compatibility of the PPy matrix, and hence phase separation is minimized. Much efforts have been devoted for the synthesis of graphene‐PPy composites.^409^, ^410^ Flexible graphene‐PPy films are prepared by pulse‐electropolymerization of PPy on graphene surface and exhibits enhanced capacitance.^411^ Ppy is electropolymerized onto graphene surface that is attached to a titanium metal substrate via electrophoretic deposition method.^412^ The highly capacitive electrode has yielded high values of specific capacitance of 1510 Fg^–1^. The as‐prepared graphene‐hollow PPy nanoarchitecture can deliver an amazing specific capacitance at a current density of 5 A g^–1^ due to the tailored nanoarchitecture and synergetic effect between graphene and PPy.

**Figure 20:**
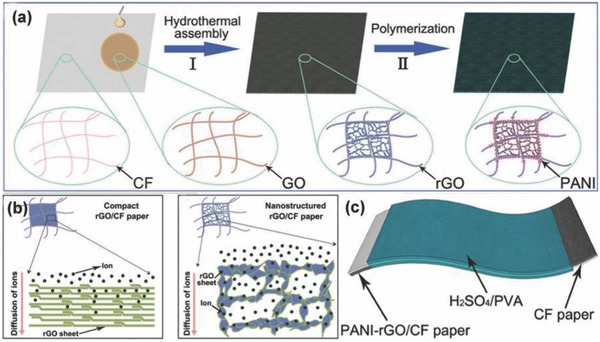
a) The preparing process of PANI‐rGO/CF composite paper. b) Illustration of ion diffusion in compact rGO/CF composite paper and nanostructured rGO/CF composite paper. c) Diagram of a solid‐state SC based on PANI‐rGO/CF paper. Reproduced with permission.^413^

For instances, Xie et al. have assembled a solid‐state SC based on PANI‐graphene/carbon fiber paper.^413^ Schematically process have been illustrated in **Figure** **20**a. At first, graphene/carbon fiber paper (rGO/CF) is obtained by uniformly coating graphene on carbon fiber paper (CF) through a “dipping and drying” method and then nanostructured graphene/carbon fiber paper is synthesized after a hydrothermal process with microporous networks. PANI is deposited onto the surface of graphene/carbon fiber paper to form PANI‐graphene/carbon fiber paper (PANI‐rGO/CF) through a chemical polymerization process. Figure 20b display the ion diffusion in compact rGO/CF composite paper and nanostructured rGO/CF composite paper, indicating that such nanostructured rGO composite paper fully utilizes the CF network and the porous structure in paper to reduce the aggregation of graphene sheets. Consequently, it facilitates not only the transportation of electrons but also the penetration of liquid ions. Solid‐state SCs are assembled using PANI‐rGO/CF as flexible electrodes which is shown in Figure 20c and the resultant device exhibit a high specific capacitance of 464 F g^–1^ due to the synergistic effect of the composites. Besides, three‐dimensional PANI‐graphene/CNT composite have been prepared via in situ polymerization and assembled into two‐electrode solid‐state SCs in PVA/KOH electrolyte. Enhanced properties are displayed with high specific capacitance of 890 F g^–1^, and good cycling stability after 1000 cycles due to the synergistic effect.

CNTs can directly serve as an outstanding scaffold to support electrochemically active due to its excellent conductivity.^414^ Composites of conducting polymers with CNTs have been proposed as the most effective solution to improve the mechanical and electrochemical properties of electrodes. Based on the electrostatic forces and p‐p stacking interactions, PANI is in situ polymerized on the surface of CNTs with special hierarchical structure.^415^ Electrochemical studies demonstrate an improved specific capacitance of 568 Fg^–1^ and stability 1 m H_2_SO_4_ aqueous solution. Flexible electrode have been fabricated by a simple in situ electrochemical polymerization of PANI on the surface of SWCNT film.^416^

Conjugated polymers are widely explored for smart materials for stretchable and transparent energy devices.^417^, ^418^ Peng group have developed a smart and stretchable electrochemical capacitors constructed of depositing PANI onto aligned CNT sheet with high performances.^419^ Aligned structure in the composite electrode facilitates the penetration of electrolyte ions. These supercapacitors exhibit high specific capacitances of 308.4 F g^–1^, and maintain a high stability after bending and stretching. Large‐scale transparent and flexible electronic devices have been fabricated using conducting polymers/CNT composite films as electrodes.

In addition to graphene and CNTs, other carbon materials are also alternatives for composites. In terms of cellulose substrates, their composites electrodes with conducting polymers have obtained higher specific capacitance than bare conducting polymers.^420^ Due to the widespread availability, low cost, and significant mechanical strength as well as flexibility, cellulose substrates are particularly appealing. The addition of cellulose significantly reduces cell resistance of these devices and then improves the electrochemical performance of PPy‐based electrodes.

Conducting polymer hydrogels are a unique class of materials that synergize the advantageous features of hydrogels and organic conductors.^421^, ^422^ Nanostructured conducting polymer hydrogels possess large surface area and three‐dimensional continuous conducting framework that can provide a relatively short diffusion path for electrolyte ions to access the electroactive surface. In that case, properly engineered nanostructure PANI/graphene hydrogel exhibit a combination of high capacitance, excellent rate performance and long cycling life.

In conclusion, metal oxides and graphene‐like layered metal compounds have been explored extensively to design electrode materials with controlled nanostructures and construct novel energy storage devices with excellent performances. Conducting polymers are a new class of electro‐active materials with low cost that exhibit both the electrical double layer derived from the surface electrostatic reaction and pseudocapacitive charge storage mechanisms resulted from the facile electron transition along the polymeric π‐conjugated chains. Especially, polyaniline and polypyrrole are widely used as cooperative materials for other electro‐active materials to enhance their capacitive behaviors.

## 4 New‐Concept SCs

### 4.1 Microsupercapacitors

Microsupercapacitors (MSCs) have gained special attention as an emerging candidate microscale energy source to keep up with the fast evolution of portable electronic devices and microelectromechanical systems.^423^, ^424^ Compared to conventional SCs, there are three chief advantages for MSCs. Firstly, micro‐size devices shorten the diffusion path lengths of the electrolyte in the normal and parallel direction of electrodes, leading to more effective utilization of the electrochemical surface area. Secondly, MSCs show higher specific capacitance, higher power delivery and knee frequency compared to conventional SCs. Thirdly and most significantly, MSCs can be integrated into various flexible and/or stretchable chips, and in series or parallel, to improve the output potential and/or current. It could potentially increase the density of supercapacitor devices and reduce their complexity by removing intricate interconnects to bulky energy storage devices.^425^, ^426^, ^427^ The possibility of integration as microelectronic devices enable them more application in integrating energy conversion devices and other electronic circuits. MSCs are expected to play an important role in future self‐powered microelectronics and microelectromechanical devices with high energy, power density and long cycle life as well as the adaptability to various substrates.

**Figure 21:**
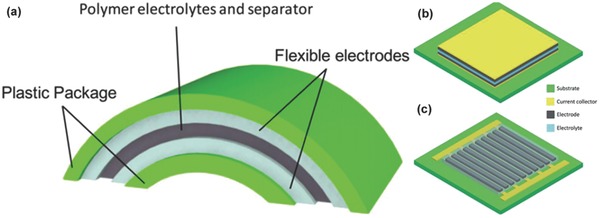
a) Schematic illustration of a flexible, solid‐state supercapacitor device.^430^ Reproduced with permission. Copyright 2014, RSC. b) Sandwich MSCs. c) Planar MSCs. Reproduced with permission.^438^ Copyright 2014, RSC.

Recent boom in all‐solid‐state SCs has stimulated the development of MSCs. Solid‐state SCs feature many important advantages including light‐weight, flexibility, ease of handling and a wider range of operating temperatures.^428^, ^429^ The use of solid‐state can avoid leakage of electrolyte, thus reduce the cost of device packaging. Furthermore, all‐solid‐state SCs can be directly implemented in portable nanoelectronic systems. Generally, the configuration of solid‐state SC devices is similar to those of conventional SCs, which are constructed of flexible electrodes, solid‐state electrolyte, a separator and a flexible packaging material as illustrated in **Figure** **21**a.^430^ The performance of solid‐state SCs are largely dependent on the electrical properties and structures as well as mechanical integrity of electrode materials and the design of suitable configuration. The realization of highly flexible and solid‐state energy‐storage devices strongly depends on both the electrical properties and mechanical integrity of the constitutive materials and the controlled assembly of electrode and solid electrolyte.^431^, ^432^

The performances of MSCs are largely dependent on the intrinsic properties of electrode materials, the electrolyte, architectural design of the device and the fabrication methods.^433^, ^434^ On one hand, the electrode materials are critical to energy storage capability for MSCs. Their electrode materials are required to possess good electrical and mechanical properties to accommodate large levels of strain without sacrificing performance.^435^, ^436^ Recently, intensive notable advancement have been focused on the fabrication and optimal design of flexible electrodes. Electrode materials with proper nanostructures would play an important role in the power density and the cycling stability. A series of nanostructured electrode materials have been developed including carbon‐based materials, metal compounds and conducting polymer, etc. It is urgent to develop ultrathin, flexible electrodes for MSCs.^437^ Hierarchical structures have been engineered with large surface to‐volume ratios to provide high energy and power densities.

On the other hand, configuration design is crucial for determining the energy density within a specified mass or volume of storage material. Figure 21b and c illustrate the two main types of MSCs with different structures.^438^ Figure 21b illustrates the conventional sandwich configuration. Stacked design with sandwich structure is assembled by two film electrodes with a small amount of gelled electrolyte which also acts as the separator. However, sandwich MSCs are incompatible with integrated circuits.

The recent boom in miniaturized electronic devices has increased the demand for MSCs which are compact and easy to be integrated into on‐chip energy storage devices.^439^, ^440^ Planar MSCs have been considered as competitive candidates for integrated energy devices and systems.^441^ Figure 21c describes the planar configuration which is consisted with interdigitated microelectrodes. Planar MSCs are in‐plane designs with confined thickness in the vertical direction and extensions along the two‐dimensional horizontal planes, which make compact device design possible. In general, planar MSCs require electrodes with two‐dimensional permeable channels. Since 2003, Sung et al. reported planar MSCs based on conducting polymers for the first time.^442^ Tremendous attention has been focused on planar MSCs and the fabrication onto a chip. The fabrication methods involve a number of printing and lithography techniques or employ masks for the definition of patterns on substrates.

Planar MSCs have several advantages. Firstly, two electrodes are arranged in the same plane, which is compatible with on‐chip integration. On‐chip integration can drastically increase the density of supercapacitor devices. Secondly, the diffusion distance for ions transport becomes shorter and can be controlled for optimizing kinetic performance. Planar MSCs are beneficial for achieving high ratio of energy delivery at high charge‐discharge rates. Thirdly, two electrodes are isolated electronically by physical separation and no separator is necessary for device construction, which reduce the design complexity by removing intricate interconnects to bulky energy storage devices. Fourthly, the interdigitated structure can potentially be extended to three dimensional devices, thus more active materials are available in the electronic circuit.

#### 4.1.1 Sandwich MSCs

Sandwich MSCs composed of thin‐film carbon electrodes are expected to possess high capacity while maintaining light weight and flexibility.^443^, ^444^ Carbon films serve as both electrodes and charge collectors which eliminates the metal‐carbon charge collector‐electrode interface, leading to a simplified and lightweight architecture. The travel distance of electrolyte ions in thin‐film SCs is also shorter than their counterparts.^445^

CNTs‐based thin films have been explored as electrode materials for sandwich MSCs. Thin film fabricated from sprayed networks of SWCNTs shows very high energy and power densities.^446^ For instances, Niu et al. have developed highly stretchable buckled SWCNT films as MSCs electrodes.^447^ The stretchable buckled SWCNT films are achieved by combining SWCNT films with continuous reticulate architecture with polydimethylsiloxane with enhanced prestrain. The resultant MSCs exhibit nearly unchanged performances even under the strain of up to 140%.

Monolithic carbon films derived from CDCs are interesting materials for MSCs due to the thin and thick uniform films and low elevated temperature.^448^ CDCs films on conductive TiC substrates have demonstrated their potential application in MSCs with exceeding volumetric capacitance. It is worth noting that volumetric capacitance will decrease along with the increasing coating thickness. The volumetric capacitance of as‐prepared microscale device can achieve nearly 180 and 160 F cm^–3^ in organic and H_2_SO_4_ electrolyte at the coating thickness of 2 um.

Graphene films have been synthesized by CVD method with superior flexibility and strength.^449^ The as‐prepared devices display remarkable electrochemical performances including high area specific capacitance of 8 mF cm^–2^, excellent rate capability and cycling performance with 100% of capacitance retention after 1000 charge‐discharge cycles.

#### 4.1.2 Planar MSCs

Most recently, graphene and CNTs are explored for the construction of planar MSCs with superior electrochemical properties.^450^ Planar MSCs based on stretchable SWCNT electrodes display high performances with a good capacitance of 100 µF and good cycle stability even under bending.^451^ Liu et al. have exploited graphene quantum dots as negative active material and PANI as positive active material for an asymmetric microsupercapacitor.^452^ Graphene quantum dots are suitable as MSCs electrode materials due to their novel chemical/physical properties such as nanometer‐size, abundant edge defects, good electrical conductivity and better surface grafting.^453^ Desirable electrochemical capacitive performances are revealed including excellent rate capability, faster power response capability and better cycling stability.

**Figure 22:**
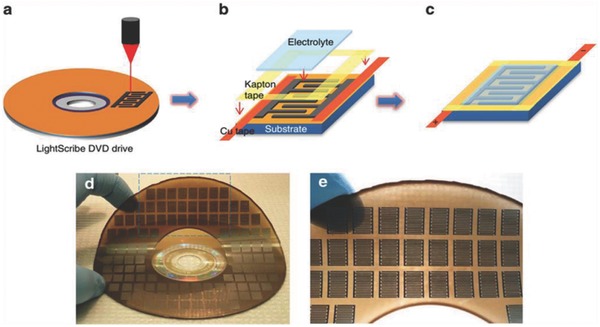
Schematics of laser‐patterning of hydrated GO films to fabricate planar microsupercapacitor devices. Reproduced with permission.^457^ Copyright 2013, Nature Publishing Group.

Laser writing is demonstrated as one effective and scalable fabrication of planar SCs based on graphene films. This direct ‘writing’ technique is low‐cost and simple post‐processing. Singh et al. have fabricated in‐plane monolithic SCs by laser reduction and patterning of GO films.^454^, ^455^, ^456^ **Figure** **22** has schematically demonstrated the fabrication process of graphene planar MSCs through the direct laser writing.^457^ As shown in Figure 22a, a DVD media disc is exploited as a substrate to support a GO film on a PET sheet. The GO film is converted into black graphene to produce interdigitated graphene circuits with the help of the laser inside the LightScribe DVD drive. Planar MSCs are created after additional process with polyimide (Kapton) tape and copper tape as well as the addition of ionogel electrolyte as shown in Figure 22b and c. Figure 22d and e illustrate that the technique is potential for the direct writing of micro‐devices with high areal density. More than 100 micro‐devices can be produced on a single run. The energy and power capacities extracted from the micro‐devices can be controlled by varying the dimensions of the interdigitated micro‐electrodes per unit area. The as‐prepared planar SCs displays an ultrahigh power of 200 W cm^–3^ and excellent frequency response with an RC time constant of only 19 ms.

Lateral ultrathin graphene interdigitated microelectrodes have been prepared by combining photolithography with selective electrophoretic buildup.^458^ Nitrogen and boron co‐doped graphene films have been assembled into planar devices through lithographically dry‐etching the films on silicon wafers.^459^ MSCs based on these interdigitated electrodes deliver the enhanced capacitances by the geometrical area and volume.

Graphene and CNTs composite have been exploited to construct planar MSCs by a simple layer‐by‐layer approach and photolithography lift‐off approach.^460^, ^461^ The presence of CNTs layer can reduce the agglomeration of graphene nanosheets and enhance the permeation of electrolyte. Consequently, the electrochemical performance can be improved by offering higher output voltage and current production.

In addition, Simon et al. have developed onion‐like carbon and carbide‐derived carbon as microsized electrodes. Onion‐like carbon particles are deposited onto interdigital gold current collectors patterned on silicon wafers by an electrophoretic deposition technique.^462^ Microdevice assembled by the well‐defined pattern exhibit an excellent cycling performance. Monolithic carbide‐derived carbon films are formed as MSCs electrode by selectively etching metal from titanium carbides substrates which have been sputtered on the SiO_2_ surface of a silicon chip.^463^ Electrochemical studies reveals their volume capacitances decreases with increasing coating thickness.

Recent progress have been achieved on developing pseudocapacitive materials on planar MSCs.^464^ Metal oxide/hydroxide are used for the construction of on‐chip MSCs with high performance.^465^, ^466^, ^467^ Chu et al. have fabricated MSCs based on nickel and cobalt hydroxides thin films employing silicon microchannel plates with a large surface area as substrates.^468^ MnO_X_/Au multilayers deposited on polyethylene terephthalate substrates are endowed with high volumetric capacitance of 32.8 F cm^–3^, a maximum energy density of 1.75 mWh cm^–3^ and a maximum power density of 3.44 W cm^–3^ as well as an impressive long‐term cycling stability.^469^

Graphene‐like layered metal compounds such as MoS_2_ has been developed to construct practical power sources for the next‐generation intelligent devices. For instances, Cao et al. have fabricated MSCs well defined by ten interdigitated electrodes on a large scale via simple painting of MoS_2_ nanosheets on Si/SiO_2_ chip to form thin films and subsequent laser patterning.^470^ The optimized MoS_2_‐based MSCs exhibits excellent electrochemical performances with a high area capacitance of 8 mF cm^–2^ and a volumetric capacitance of 178 F cm^–3^ as well as outstanding cyclic performance in aqueous electrolytes.

PANI is a favourable choice for planar configuration because of its low cost, ease of synthesis, relatively high theoretical capacity, and flexibility. For example, Yuan et al. have constructed a highly flexible and high‐performance MSCs using PANI/Au/paper structures been employed as electrode materials.^471^ Wei et al. have designed a pattern of PANI nanowire arrays via in situ chemical polymerization method and applied as microelectrode for flexible MSCs on a chip by micro‐fabrication technology.^472^

**Figure 23:**
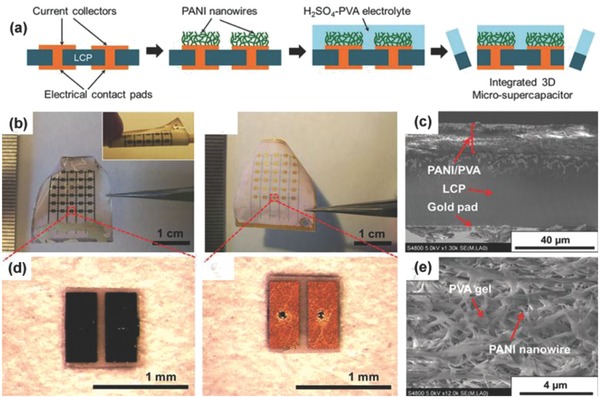
a) Fabrication of integrated three‐dimensional MSCs. b) Photographs of the topside and backside of an LCP sheet. c) Cross‐sectional SEM images of the whole device. d) The vertical stack‐up of the backside pad, host LCP substrate, and PANI electrode bonded with H_2_SO_4_‐PVA electrolyte. e) SEM image of PVA and PANI nanowire. Reproduced with permission.^473^

Moreover, a novel integrated MSCs technology with three dimensional micro‐integration capabilityhave been developed based on PANI by through‐via bottom electrode contact.^473^ As illustrated in **Figure** **23**a, liquid crystal polymer (LCP) sheets and gold current collectors are used as substrates for the growth of PANI nanowires. A single microsupercapacitor is fabricated by as‐prepared PANI free‐standing electrodes using PVA‐H_2_SO_4_ polymer electrolyte as electrolyte and separator. Three dimensional MSCs are integrated after embedding the entire electrical routing path and contact pads within the effective device area by through‐via bottom electrode contact. Figure 23b–e show the graphs of as‐fabricated integration of multiple MSCs and the single devices. Figure 23f determined the cross‐sectional morphology of the whole device indicating the thickness of PANI, LCP and gold pad. PANI nanowires and PVA gel can be clearly recognized in Figure 23g. Attractive properties have been tested by electrochemical measurements with high areal/volumetric capacitance and energy and power densities. Three‐dimensional MSCs would solve the problems that the limited surface area is available in the electronic circuit and allow for high‐power energy storage. This approach constitutes a promising way for the development of conducting polymer‐based electrode materials for integrated MSCs.

### 4.2 Fiber SCs

Increasing power and energy demands have spawned the emergence of fiber SCs for next‐generation portable, stretchable, and/or wearable energy storage devices.^474^, ^475^, ^476^ In fiber SCs, two fibers are arranged either helically or in parallel. The devices made in a wire or fiber format, they can be lightweight and flexible and easily folded, rolled up, woven into textiles or even reshaped into other architectures with low cost and high efficiency. In recent years, extensive efforts have been devoted to explore stretchable fiber electrodes with a high elasticity and good conductivity. High‐performance fiber SCs based on carbon nanomaterials, metal compounds nanowires, or conductive polymers have been developed to meet the growing demand for flexible and wearable electronics.^477^, ^478^

**Figure 24:**
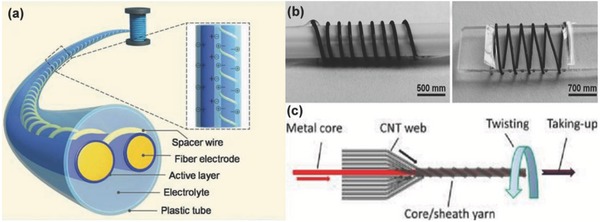
a) Architecture of the fiber SCs and morphology of the electrodes. Reproduced with permission.^482^ b) Aligned CNTs are wound on elastic substrates. Reproduced with permission.^483^ c) Aligned CNTs are wound around metal core. Reproduced with permission.^487^ Copyright 2014, ACS.

Firstly, carbon‐based fiber has been explored in flexible energy‐storage electrodes due to its unique features involving the high flexibility, good mechanical properties and its unchanged sheet resistance even in a very high bending state. CNTs have been studied widely as fiber electrode materials in energy storage devices due to the unique structure and remarkable properties including high flexibility, tensile strength, electrical conductivity, as well as mechanical and thermal stability.^479^, ^480^, ^481^ The rapid charge separation and transport can be expected due to the unique one‐dimensional structure of CNTs with high electrical conductivity. Conventional CNTs‐based fiber electrodes are composed of conductive fibrous substrates and electrochemically active CNTs materials. **Figure** **24**a shows the structural diagram of a flexible fiber SCs constructed of two fiber electrodes, a helical spacer wire and an electrolyte.^482^ A spacer wire is used to effectively prevent short circuits. The fiber electrodes are placed closely parallel and packaged into a flexible plastic tube filled with electrolyte.

In general, the elastic polymers are used as the substrates to enhance the flexibility. Peng group have wrapped aligned CNTs on elastic substrates as two electrodes as shown in Figure 24b.^483^, ^484^ At first, spinnable MWCNT arrays are synthesized by CVD method. Aligned MWCNT fibers are prepared by spinning from the array and then used as active materials. The aligned MWCNT fibers are wrapped around elastic wires and fiber electrodes are made by twisting two such CNT‐wrapped elastic wires.^485^ The resultant wire‐shaped SCs exhibit a high device capacitance and elasticity up to 350% strain.^486^ Moreover, no change has been observed after repeatedly stretching from 0 to 200% strain for hundreds of cycles. On the other hand, a highly conductive metal filament core has been embedded in the center of a CNT yarn for the fabrication of core/sheath structured CNT yarn electrodes, as illustrated in Figure 24c.^487^ Metal wires serve as current collectors to improve the electrical conductivity. The highly conductive core provides an efficient channel for charge transportation, which result in significant improvement of electrochemical performances.

Additionally, flexible fibers and yarns based on graphene are attractive as electrode for fiber SCs. For example, Wallace et al. have developed an optimized commercially viable, simple wet‐spinning route, followed by an optimal heat‐treatment regime to obtain graphene yarns with a high interlayer spacing in between graphene sheets.^488^ Shi et al. have deposited graphene on gold wires by an electrochemical deposition method.^489^ Graphene ribbons have been explored for SCs applications.^490^ The resultant graphene yarns exhibit extraordinary capacitance, rate capability, long cycling life and attractive flexibility. Moreover, graphene can be incorporated with CNTs to improve the charge transport resulted from strong π‐π interactions and dispersibility. CNT/graphene fibers by biscrolling method have high electrical conductivity and tensile strength, which is appropriate to fabricate flexible SCs with a high specific capacitance up to 4.97 mF cm^–2^.^491^ The wire‐shaped SCs assembled based on CNT/graphene fibers by wet‐spinning and reduction process presented a high volumetric capacitance of 38.8 F cm^–2^.^492^

In addition, mesoporous carbon particles and carbon microfiber are bundled onto the alignment of CNTs to produce fiber electrodes.^493^, ^494^ The presence of mesoporous carbon favors a rapid charge separation and transport and accessible to the electrolyte solution resulted from the high specific surface area and pore structure. Consequently, a high specific capacitance of 39.7 mF cm^–2^ is achieved.^495^ Nitrogen‐doped activated nanofiber derived from pyrolyzed bacterial cellulose have been obtained by a hydrothermal reaction and used as active materials for fiber electrodes.^496^ Good supercapacitive performances have been revealed including a maximum power density of 390.53 kW kg^–1^ and a superior cycling stability after 5,000 cycles.

Secondly, there have been some reports on metal oxide/hydroxide/sulphide fiber SCs.^497^, ^498^, ^499^ The fiber electrodes exhibit outstanding mechanical robustness and electrochemical performances. For example, hierarchical ZnCo_2_O_4_ nanowire arrays/carbon fibers have been synthesized and further used as electrodes for fiber SCs.^500^ MnO_2_ deposited onto three‐dimensional nickel nanocone arrays have been employed as electrode to fabricate ultrathin and flexible fiber SCs.^501^ Moldovan et al. have demonstrated a cylindrical metal‐insulator‐metal fiber SCs dependent on Cu—Cu_2_O—C coaxial nanowires synthesized.^502^ Remarkable capacitance of 140 F cm^–2^ is observed due to the excellent thermal stability, high electrical conductivity and current‐carrying capacity. A general strategy has been developed to fabricate ultralong hybrid microfibers based on graphene and MoS_2_. The presence of MoS_2_ introduces large pseudocapacitance and serves as spacers to preserve the stacking of graphene sheets.^503^ In this case, the performance of fiber‐based solid‐state SCs was greatly improved due to the synergetic effects.

**Figure 25:**
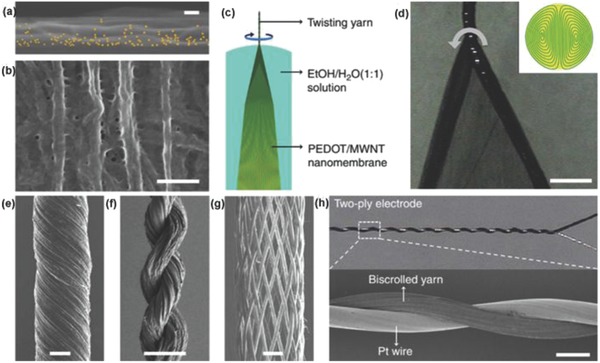
a) An EDAX (energy‐dispersive analysis by X‐ray) map of the cross‐section of a PEDOT‐infiltrated two‐layer CNT sheet stack. Scale bar, 50 nm. b) SEM image of the side of a PEDOT‐coated MWNTs nanomembrane. Scale bar, 200 nm. c) Schematic illustration showing the fabrication of a biscrolled PEDOT/MWNT yarn. d) Optical microscope image of the spinning wedge, which shows the wedge edges being twisted to form a dual‐Archimedean scroll yarn, which is schematically illustrated in the inset. Scale bar, 200 mm. e) SEM image of a biscrolled yarn with bias angle. Scale bar, 10 mm. f) SEM image of two PEDOT/MWNT yarns plied together. Scale bar, 50 mm. g) SEM image of a braided structure containing 32 biscrolled yarns. Scale bar, 100 mm. h) Two SEM images of a PEDOT/MWNT biscrolled yarn that is plied with a 25 mm Pt wire. Scale bar, 40 mm. Reproduced with permission.^506^ Copyright 2013, Nature Publishing Group.

Thirdly, conducting polymers are well known to be useful as the active material for fiber SCs electrodes owing to their conducting properties and charge storage based on both Faradaic and non‐Faradaic process.^504^, ^505^ Flexible and weavable PEDOT‐MWCNT yarns have been fabricated through a biscrolling process and developed as high performance electrodes for MSCs.^506^ At first, PEDOT are deposited onto MWNT by vapor phase polymerization and then the PEDOT location and the side of PEDOT‐coated MWNTs nanomembrane are determined in **Figure** **25**a and b. Figure 25c illustrated the biscrolling process for PEDOT/MWNT yarns for which twist insertion are achieved by using a rotational electric motor under the help of an ethanol/water solution. Afterwards, a wedge structure is formed as the nanomembrane is twist spun into yarn in Figure 25d. Biscrolling process generated bias angel between the yarn direction and the orientation direction. The high mechanical strength and flexibility of biscrolled yarns enabled fabrication of two‐ply yarns and 32‐yarn braids (Figure 25e–g).

A Pt wire is used as current collectors to increase power generation capabilities and provide fast ion‐transportation. A biscrolled PEDOT/MWNT yarn was plied with current collector to fabricate the identical electrodes used as both anode and cathode (Figure 25h). A solid stated fiber supercapacitor is conducted using PVA‐H_2_SO_4_ as electrolyte and a high volumetric capacitance up to 179 F cm^–3^ are exhibited. Moreover, extraordinary high power and high energy storage capabilities and high cycle life make them promising applications in electronic textiles. Composite yarns based on polymer and carbon or/and metal oxide have been prepared by a number of approaches and exhibit advanced electrochemical performances.^507^, ^508^, ^509^ Fiber SCs based on CNTs and PANi composites have superior properties to those based on pure CNTs. Moreover, the as‐prepared yarns can be woven into wearable electronics due to their excellent mechanical performance.^510^

### 4.3 Li‐/Na‐ion Capacitors

#### 4.3.1 Li‐Ion Capacitors

Li‐ion capacitors (LICs) as one of hybrid SCs have attracted tremendous research interests as advanced electrochemical energy storage devices in recent years. LICs are generally constructed by a negative electrode that can be doped with Li^+^ (battery‐type electrode) and an activated‐carbon positive electrode (capacitor‐type electrodes) and Li^+^‐based nonaqueous electrolytes. They feature higher charge and discharge rate than lithium ion batteries, and higher output potential difference than EDLCs.^511^, ^512^ Besides, LICs deliver lower self‐discharge rates than EDLCs and moderate thermal behavior.^513^ Extensive researches have been done for advanced improvement for LICs application.

The positive (cathode) electrode made of activated carbon(AC) is dependent on EDLCs charge mechanism.^514^ Wang et al. have assembled LICs with tailored capacity using AC cathode material. Further research reveals that optimized electrochemical performance of LIC can be realized via the positive electrode capacity design.^515^ To construct high energy density LIC, high surface area porous carbon have been synthesized as a cathode material. For instance, AC with controllable mesoporosity has been prepared from coconut shells by both physical and chemical hydrothermal carbonization activation process.^516^ The LICs display a maximum specific capacitance of 159 F g^–1^ and high energy density of 69 Wh kg^–1^ in non‐aqueous medium. Carbons derived from MOF are featured with three‐dimensional architectures.^517^ Hence, enhanced electrochemical properties are investigated with outstanding cyclability and high energy density without compromising the power capability.

Graphene is a very promising candidate as cathode material of LICs due to the high specific surface area and good electronic conductivity.^518^, ^519^ Ruoff groups have applied chemically activated graphene as cathode material for LICs.^520^ A hybrid cell is constructed using chemically activated graphene and Li_4_Ti_5_O_12_ as cathode and anode materials, respectively. Superior properties are investigated with an excellent gravimetric energy density for a packaged cell of 53.2 Wh kg^–1^ at operating potentials of 4 V.

To improve the energy density, Li_2_RuO_3_ is chosen as a new additive due to its highly reversible characteristics and structural stability.^521^, ^522^ The addition of Li_2_RuO_3_is favourable for improved electrochemical performances and safety of LICs. The superior performance is resulted from the synergy effect between the active materials and the additive.

The negative electrode material is a key research to the development of LICs.^523^ The charge storage for the negative (anode) electrode is resulted from a reversible Faradaic reaction of lithium ion insertion/extraction. Generally, the LIB anodes have been utilized in LICs. The promising negative electrode materials can be divided into three categories: carbons with lithium pre‐doping, metal oxides (TiO_2_, V_2_O_5_, etc.) and spinel family materials such as Li_4_Ti_5_O_12_, LiCrTiO_4_ and so on.

Firstly, carbons with lithium pre‐doping stand their point for negative electrode because of their unique properties. The effect of pre‐lithiation degree has a dramatic effect on the stability of the negative electrode and cycling stability. The appropriate pre‐lithiation ensures the low working voltage of negative electrode and relative stable charge‐discharge platform.^524^, ^525^ Various types of carbons have been evaluated as negative electrodes for LICs, which are classified into graphite, non‐graphitizable carbon (hard carbon) and graphitizable carbon (soft carbon)based on theircrystallinity.^526^ Their electrochemical performances have been in‐depth studied as negative electrode for LICs.

Hard carbon has attracted more attention for their reasonable synthesis temperature and excellent cycleability and high input/output performance. LICs have been developed using a mixture of stabilized lithium metal powder and hard carbon as the anode electrode. When LIC is constructed by hard carbon as anode and AC as the cathode, it exhibits an outstanding cycleability with less than 3% degration over 600 cycles.^527^ A detailed study of hard carbon anode has been conducted on the effect of with different loadings of stabilized lithium metal powder and voltage drop.^528^ It was found that smaller voltage drop was delivered by LICs with low loadings of stabilized lithium metal powder.

In general, soft carbon has been represented as a promising alternative for anode materials with high‐rate performance. Balducci group have devoted much effort on use of soft carbon as anode material. Prefer capacity retention has been found in soft carbon at high rate due to the effective lithium insertion process over the used potential.^529^, ^530^ High‐performance LICs have been realized by using soft carbon as anode material and propylene carbonate as electrolyte solvent.^531^ Superior performances have been achieved with average energy density of 21.7 Wh kg^–1^ at a current density of 5.6 A g^–1^.

**Figure 26:**
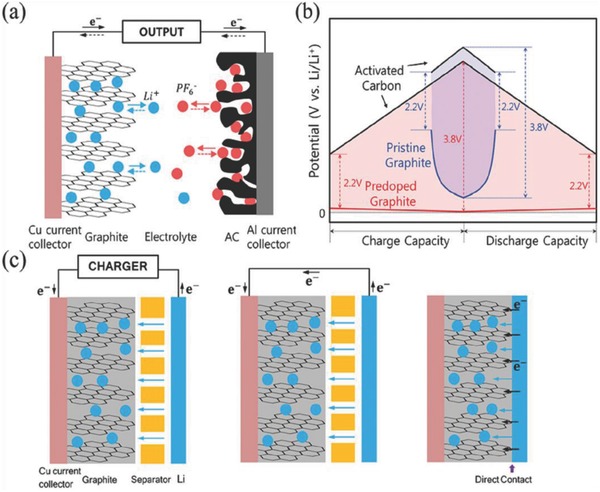
a) Schematic diagrams of the LIC composed of the activated carbon cathode and the pre‐doped graphite anode. b) Typical charge‐discharge profiles. c) Schematic diagrams of three different pre‐doping methods. Reproduced with permission.^536^ Copyright 2014, RSC.

Graphite has been applied as negative electrode for LICs. Fuji Heavy Industry has reported the LICs for the first time as an example of concept.^532^ Since then, more graphite‐based devices have been attempted and a high energy density have been realized up to 80 Wh kg^–1^.^533^ Pandolfo groups have assessed the suitability of graphite as a negative electrode in LICs.^534^, ^535^ For instance, the LIC has been composed by an AC cathode and a lithium pre‐doped graphite anode in LiPF_6_ electrolyte, as illustrated in **Figure** **26**a.^536^ During the charging and discharging processes, the charge storage on the cathode refers to the adsorption and desorption of anions whereas the anode side occurs Faradic redox reaction.

The flat discharge profile of lithium pre‐doped anode electrode results in a wider potential voltage window, which is conducive to higher energy density as shown in Figure 26b. The lithium pre‐doping process are carried out via three methods with a sacrificial lithium metal electrode as a lithium ion source in a nonaqueous electrolyte as shown in Figure 26c. The left and middle figures demonstrate the electrochemical and external short circuit method where the two electrodes are separated by a porous separator in the nonaqueous electrolyte. A long pre‐doping time is required for the sufficient and uniform lithium pre‐doping level. The right figure introduces an effective internal short approach with the large electrochemical potential difference and to the direct contacts over the wide area between the graphite and lithium electrodes. In this system, LICs can reach high working voltages up to 4V. In addition, the electrochemical behavior of Li^+^ insertion into battery‐type electrodes results in overwhelmingly large capacity.

For fabrication of lithium pre‐doped electrode, metallic lithium is widely used as lithium sources during the lithium doping process. However, metallic lithium needs careful handle because it is easy to explode under moisture environment. Besides, the long pre‐doping time increases the cost of LICs. Hence, a stable and safe lithium source is necessary to meet safety requirements for the application of LICs. To address this problem, Park et al. have proposed Li_2_MoO_3_ as an alternative lithium source to ensure the safety requirements and simplified doping process.^537^ In addition, single‐phase Li_5_FeO_4_ with an antifluorite structure has been considered as a promising alternative lithium source instead of metallic lithium.^538^ These alternative lithium source can yield not only sufficient Li^+^‐doping efficiency but also improved safety by avoiding the use of metallic lithium during the cell assembly.

Traditional metal compounds have been considered as promising insertion type electrode materials for LICs applications such as TiO_2_, VO_x_ and so on.^539^, ^540^ For instances, Fan et al. have constructed LICs by combining TiO_2_ nanobelt arrays anode and graphene hydrogel cathode.^541^ VO_x_ possess highly reversible Li‐ion intercalation/de‐intercalation behaviorand deliver outstanding energy density.^542^, ^543^ Nb‐based compounds have been developed for high‐energy‐density LICs with a high power capability. LICs constructed of TiNb_2_O_7_ nanofibers and AC deliver a maximum energy density of about 43 Wh kg^–1^.^544^ Nb_2_CT_x_/CNT electrodes show high volumetric capacitance of 325 F cm^–3^ when tested in a LIC configuration.^545^ Composites based on metal oxides and carbon materials have been developed as an anode electrode material for LICs.^546^, ^547^ Enhanced performance is expected from the synergistic effect of metal oxides and carbon materials.

Spinel family (AB_2_O_4_) materials with promising characteristics have been studied extensively as prospective insertion hosts for LICs.^548^ Among the insertion type anodes investigated, spinel Li_4_Ti_5_O_12_ is found to be superior in terms of low cost, good reversibility and no volume variation during lithium insertion/extraction.^549^ LICs based on Li_4_Ti_5_O_12_ can be modulated by tuning the cathode/anode mass ratio.^550^ Excellent performances have been realized with optimized mass loading including a maximum energy density of 19 Wh kg^–1^. However, their energy density is limited resulted from the restricted amount of lithium insertion into spinel‐phase Li_4_Ti_5_O_12_. More Li‐enriched materials have been explored as anode for LICs. For example, LiTi_1.5_Zr_0.5_(PO_4_)_3_ with a nasicon‐type structure allow insertion of 2 mol lithium at 2.5 V vs. Li/Li^+^.^551^ Carbon‐coated Li_3_V_2_(PO_4_)_3_ phase based LICs deliver a maximum energy densities of 27 Wh kg^–1^.^552^

#### 4.3.2 Na‐Ion Capacitors

The hungry demand for vast energy sources and the shortage of lithium resources on the earth make it urgent to explore alternative SCs using abundant and economic materials. In principle, resources for sodium are sufficient and very easy to renew. Sodium ion‐based energy storage devices are the most appealing alternative choice including sodium ion capacitors (NICs).^553^ NICs have been explored in recent years as a promising alternative to LICs. Analogue to LICs, NICs is fabricated by capacitor‐type positive electrode and battery‐type negative electrode in conjunction with Na^+^‐based electrolytes.

Na_4_Mn_9_O_18_ has been reported as a sodium intercalation positive electrode material for an aqueous electrolyte energy storage device. Na_4_Mn_9_O_18_ has been synthesized through a simple solid‐state synthesis route and sol‐gel method.^554^, ^555^ The NICs are assembled using Na_4_Mn_9_O_18_ as positive electrode and AC as negative electrode in 1 m Na_2_SO_4_ electrolyte. The proper mass ratio of positive and negative electrode is critical to the electrochemical performance.

The consideration for NICs has focused on the choice of satisfactory negative electrode materials to meet the demand of high energy density.^556^ Carbon can be explored as negative electrode materials by sodium pre‐doping. NICs based on hard carbon and amorphous carbon have been fabricated and show remarkable electrochemical activity.^557^, ^558^ A green and highly economical carbon has been obtained from peanut shells and applied into NICs.^559^ The as‐prepared carbon possess hierarchically porous architecture which favor the ion adsorption. It is worth noting that the resultant NICs demonstrate superior energy and power density compared to LICs within a wide temperature range.

Nonetheless, NICs have not attracted more attention as a charge carrier in past years because the larger ionic radius sodium prevents the intercalated reaction into graphite. Till 2000, Stevens and Dahn revealed that sodium can be predoped into the hard carbon to form the negative electrode, which inspired subsequent efforts on the negative electrode but also the positive electrode of NICs.^560^ The viewpoint of their low Na^+^‐insertion potential profiles make it possible that some Na^+^‐related materials may be suitable for negative electrodes. For instance, sodium titanate nanotubes have been used as negative electrode materials and exhibit excellent rate capability and desirable energy density and power density.^561^

Transition‐metal oxides have been also exploited as anode materials for the fabrication of high‐performance NICs. For example, spinel NiCo_2_O_4_ was applied into NICs with a superior performance.^562^ Layer‐structured V_2_O_5_ nanowires/CNTs composites serve as anode materials for NICs.^563^ The incorporation of V_2_O_5_ with CNTs possess porous networks allowing sodium insertion/extraction and fast electron transfer. The as‐fabricated NICs deliver a maximum energy density of 40 Wh kg^–1^ with a maximum voltage of 2.8 V. However, previous results cannot satisfy superior capacitive behaviors due to the slow kinetics of ion intercalation or small EDLC. MXene Ti_2_C has been developed as a negative electrode for NICs to deliver a higher specific capacity and rate capability.^564^

In conclusion, new‐concept SCs have been represented including MSCs, fiber SCs and Li/Na‐ion capacitors. MSCs and fiber SCs have been under the most extensive research for the development of the fabrication and packaging technique for highly‐efficient, light‐weight and portable devices. The availability of capacitive storage based on LICs and NICs are attractive and under development.

## 5 Further Applications of SCs

### 5.1 Integrated Systems with Photovoltaic Devices

SCs are energy storage devices with superiorities of high power performance, long cycle life, and low maintenance cost. However, they are generally charged by external electrical power sources. On the other hand, the photovoltaic (PV) solar energy has been regarded as a clean, widely available and preferable renewable energy supply. Solar cells has regarded as a promising technology to use renewable energies. However, the solar cells are unable to store the converted energy for further use. Therefore, it is still a challenge for PV technology to storage and effective utilization of generated energy. Increasing attentions have been attracted to fabricate integrated systems by PV in conjunction with SCs recently.^565^ Conventionally, PV devices are applied for energy harvesting, which convert solar energy to electric energy. SCs are required for energy storage, which may act as both energy reservoir and power buffer. The integrated systems can be packed as a parallel combination or a photo‐supercapacitor.

#### 5.1.1 Parallel Integration

Recently, intensive progresses have been made to directly stack a PV cell and a SC into one device which not only convert solar energy into electricity but also store electricity in the form of chemical energy. Compared with single energy devices, integrated power devices can achieve higher energy conversion efficiency and energy storage density so that avoid energy wastage and maintain system stability. Consequently, integrated power devices are extremely important for further self‐driven systems.

A parallel package is composed by the combination of two separate devices with two compartments, a solar cell and a capacitor. An external circuit, such as a diode switch, is required to connect the solar cell to the capacitor during energy harvesting cycle and prevent the capacitor discharge through the solar cell. Integrated power devices have been introduced by incorporating dye‐sensitized solar cells (DSSCs) with a semiconductive metal oxide‐based SCs.^566^ Metal oxides have been used as planar electrode materials for the integration systems such as TiO_2_, SnO_2_ and so on.^567^, ^568^, ^569^ The integration systems permit the direct storage of energy generated by sunlight within single optoelectronic microelectrochemical device.

Conventional electrodes cannot satisfy the development of flexible and weaveable devices in modern electronics. As a result, it is still a challenge to develop a highly flexible and portable integrated energy package. Some attempts have been devoted to fabricate wire‐shaped DSSCs and they can be further integrated with SCs to simultaneously realize the flexible energy conversion and storage. Twisted fiber‐like electrodes have explored to construct the integrated power fiber system such as fibers based on modified Ti wire/CNT and ZnO nanowires/graphene.^570^, ^571^ Peng group have developed a self‐powered “energy fiber” for integrated systems. The “energy fiber” is flexible and can be scaled up for the practical application by the well‐developed textile technology, and may open a new avenue to future photoelectronics and electronics.^572^

**Figure 27:**
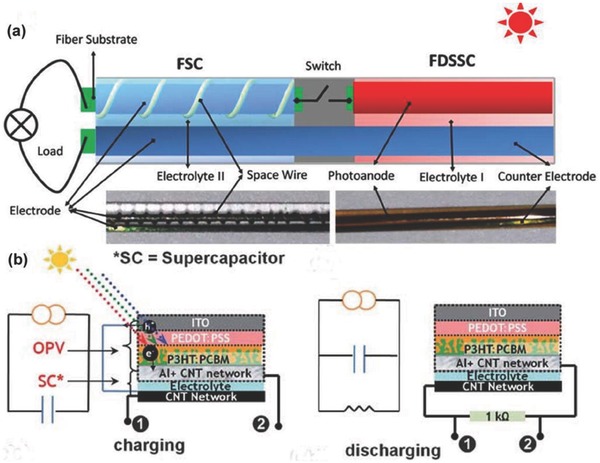
a) Structural schematic and photograph of an integrated power fiber consisting of dye‐sensitized solar cells (FDSSC) and fiber SCs (FSC). Reproduced with permission.^573^ Copyright 2013, RSC. b) Schematic and circuit illustration of a photo‐supercapacitor (PSC) during the charging (left) and discharging process (right). Reproduced with permission.^576^ Copyright 2011, RSC.

An integrated fiber power device has been illustrated in **Figure** **27**a.^573^ A stainless steel (SS) wire coated with PANi film is employed as the counter electrode of the fiber dye‐sensitized solar cell (FDSSC) and the electrodes of the fiber supercapacitor (FSC). On one hand, a space wire was wound around the PANi/SS electrode of FSC to prevent a short circuit. Electrolyte II is 1 m H_2_SO_4_ solution which serve as electrolyte for FSC. On the other hand, FDSSC is composed of a counter electrode (PANi/SS fiber) and a photoanode (TiO_2_‐Ti wire). Electrolyte I is used as electrolyte for FDSSC which is consisted of 0.6 m 1‐butyl‐3‐methylimidazolium iodide, 0.025 m iodine, 0.3 m 4‐tertbutylpyridine, 0.05 m lithium perchlorate and 0.05 m guanidinium thiocyanate in acetonitrile solution. For energy conversion, the photoelectric conversion efficiency of PANi‐based FDSSC can reach up to 5.41%. For energy storage, an area‐specific capacitance of FSC can reach 3 mF cm^–2^ to 41 mF cm^–2^. Besides, the overall energy conversion of the integrated system is up to 2.1%. Above all, it is successfully realized that the fiber dye‐sensitized solar cell drive the fiber supercapacitor in one device under solar exposure.

#### 5.1.2 Photo‐Supercapacitors

Photo‐supercapacitors (PSC) have emerged as integration systems by eliminating the external interconnects between the PV and SC device.^574^, ^575^ Different from the parallel integration, PSCs are operated in three‐electrode mode composed by photoelectrode, intermediate electrode and external counter electrode. The intermediate electrode is bifunctional and shared between the capacitor and solar cell, which serve for the DSC energy regeneration on one side and for the energy storage on the other side.

There are several advantages for PSCs compared to conventional parallel integration. Firstly, the energy conversion and energy storage devices are always operated independently in conventional parallel integration, which requires external electrical interconnections between the PV and SC device. Consequently, the integration systems result in energy loss and design complexity. Such an integrated device optimizes the energy utilization efficiency and reduces device volume which is beneficial for smart energy storage and compact electronic devices. Secondly, such an integrated device with a simplified architecture is compatible with the roll‐to‐roll printing and coating technologies on flexible substrates. Thirdly, an improved performance can be expected due to the reduced internal resistance and smaller voltage drop. Fourthly, enhanced energy density can be achieved due to their higher maximum voltage. Fifthly, shorter time is required for the saturation of voltage to complete charging. The feasibility of self‐charging PSCs enable them potential candidate for flexible and weaveable devices.

A printable all solid‐state PSC has been demonstrated by incorporating organic photovoltaics (OPVs) with SC using a layer‐by‐layer device fabrication approach.^576^ CNTs are explored as both of photoactive and electroactive materials due to their high electrical conductivity, chemical stability, high robustness and solution processibility. SWNT network serves as a common integration platform, enabling thinner and lighter device architecture. Figure 27b shows the schematic and circuit illustration of the PSC during the charging and discharging process. The OPV is consisted by indium tin oxide (ITO), poly(3,4‐ethylenedioxythiophene) poly(styrenesulfonate) (PEDOT:PSS), a blend of poly(3‐hexylthiophene) (P3HT), [6,6]‐phenyl‐C61‐butyric acid methyl ester (PCBM)and patterned Al cathodes. The SC is fabricated using free‐standing CNT as electrode materials. In PSC, OPV and SC are integrated in parallel with a common interface. During charging process, the light source is switched on and the charging process is undertaken in which OPV harness energy and then the photogenerated charge are stored at SC. After charging, the light source is switched off and the discharging process is undertaken in the dark. Integration systems combined by OPVs with SCs offer a promising opportunity to store energy. Foy energy conversion, the OPVs presented power conversion efficiency of 3.39%. For energy storage, SC device yielded a power density of 0.15 kW kg^–1^ and an energy density of 2.96 Wh kg^–1^ by potentiostatic measurements. Hence, the devices can be used on demand for harnessing applications to provide electricity reliably for off‐grid and commercial applications.

PSCs have been constructed with enhanced photoelectric conversion and storage efficiency. An individual silicon wafer is processed into a multifunctional platform where one side is adapted as anode of the DSSC and the other side serves as an electrode for SC.^577^ Owing to the manufacturing process, the combined integration system yield an overall photoelectric conversion and storage efficiency up to 2.1%. A novel integrated PSC thin‐film has been built based on one‐dimensional anodic titanium oxide (ATO) nanotube arrays.^578^ Bi‐polar ATO nanotube arrays are exploited as electrode materials for both DSSCs and SCs. Selective plasma‐assisted hydrogenation treatment is used to improve the performances. As a result, the power conversion efficiency of DSSCs was investigated as 3.17% with a short‐circuit current density of 9.03 mA cm^–2^, and the energy density of the SC was calculated to be 6.662 × 10^−8^ Wh cm^–2^. The integrated PSCs exhibit maximum overall photoelectric conversion and storage efficiencyand maximum energy storage efficiency up to 1.64% and 51.60%, respectively. The use of conductive CNT films can also greatly improve the photoelectric conversion and energy storage.^579^ A dye‐incorporated TiO_2_ electrode was then integrated onto one of the CNT films to achieve the photoelectric conversion. And then the integrated device exhibits a high entire photoelectric conversion and storage efficiency of 5.12% due to the aligned structure and high surface area of CNTs.

### 5.2 Large‐Scale Deployment

Extensive researches have been devoted for the advancement of SCs on aspects of materials design and devices configuration. Furthermore, it is more profound to realize the large‐scale deployment of SCs for the future energy development. SCs are potential devices for substantial scale deployment due to their rapid power delivery. With times past, versatile energy storage/power supply systems are anticipated to offer wide ranges of power density and energy density to satisfy the continuously developing demands of contemporary applications.

#### 5.2.1 Industrial Equipment Markets

SCs are capable of storing and releasing energy very quickly and effectively, which enable them serve as rapidly emerging and increasingly applied technology. During past decades, SCs are currently being utilized in diverse range of future applications in industrial equipment markets. Firstly, SCs can efficiently discharge and recharge quickly and act as a complementary energy source to other energy systems which cannot repeatedly provide quick bursts of power, such as fuel cells and batteries. For further applications, SCs and batteries can be series‐connected.^580^ Future development will witness SCs become integral parts of a wide variety of hybrid energy‐storage/power‐delivery systems.

Secondly, one application is regenerative braking due to their quick response in a short time, which is used to recover power and release regenerative braking energy efficiently to assist hybrid vehicles accelerate.^581^ The increasing carbon emissions drive automotive vehicles research and development on powering by hybrid systems to reduce weight and fuel consumption. A regenerative braking system is employed which is comprised of SCs that absorb and store virtually all kinetic energy from the braking system and then provide assisted propulsion. As a result, there are several emerging applications for SCs in automotive, aeronautic/astronautic aircrafts and portable electronics industries and various portable electronics.

Thirdly, SCs can capture energy and provide burst power to assist in lifting operations.^582^ on one hand, SCs can offer rapid storage and efficient delivery of electrical energy in heavy‐duty applications. On the other hand, SCs can operate a long service life and perform well under harsh conditions, which can meet the particular demands for heavy transportation vehicles.

#### 5.2.2 Solar Electricity Storage

Many countries have been placing greater emphasis on electrical production from renewable sources such as solar since they are renewable, environment‐friendly and low cost compared to fossil fuel‐derived power. The electricity through direct conversion of solar energy via PV technology is expected in response to future global energy and environmental challenges. However, the energy converted from solar is intermittent which is not available at night and cloudy days. To address this problem, there is a requirement for a device that can store large amounts of energy and release it when it is needed.^583^ So far, there is no single energy‐storage technology can achieve the target.

Consequently, integration systems have been considered as the most potential approach to deal with the wide energy requirements of energy storage devices, which are fabricated by combinations of PV technology and SC technology. Substantial progress has been made on the integration system composed by SCs and PVs at the laboratory level as summarized in Section 5.1. During the past decades, the energy storage field has witnessed a dramatic expansion in research directed at materials that might combine solar cells for energy conversion and SCs for energy storage.^584^ SCs hold a true potential to alleviate intermittence, and make solar electricity available at night and in cloudy days. The integration systems pave a new way for the renewable energy storage. In China, the development of PV technology is booming and solar electricity has been widely used in aspect of household life.^585^

**Figure 28:**
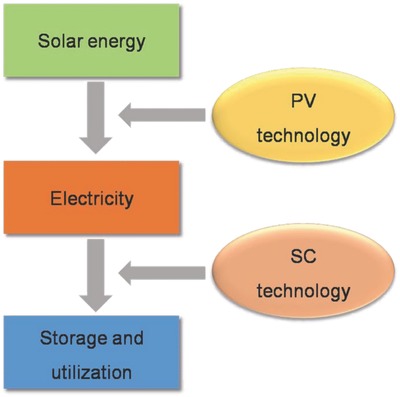
The illustration of the electricity production, storage and consumption through integrated systems by combinations PV technology and SC technology.

Integrated systems based on PV technology and SC technologies possess a lot of advantages. Firstly, the two technologies are capable to be realized due to their affordable prices for deploying in massive scale. Secondly, storing electricity produced from solar panels in SCs can be used whenever it is needed. SC technology will be vital to future clean energy storage, ensuring secure and continuous supply to the consumer.^586^ Integrated systems derived from PV technology and SC technology are currently in use in many applications such as solar home systems, solar lanterns or solar PV mini‐grids. The solar energy is converted to electricity by PV technology and then the electricity is stored in SCs and exploited by electricity‐consuming devices whenever it is needed as illustrated in **Figure** **28**. In brief, SC technology hold potential to alleviate intermittence, and make solar electricity available at night and in cloudy days.

In conclusion, integrated devices have been designed by incorporating PV devices with SCs in one device to simultaneously realize the energy conversion and storage efficiency. The PV devices harvest the solar energy and the SCs store the energy and work as a backup power system. For large‐scale deployment, SCs have been exploited across a vast swath of commercial and industrial equipment due to their unique properties including peak currents, high duty cycles and frequent deep discharge/charge cycles. Additionally, they can operate stable electrochemical performances to help ensure safety and reliability in applications. To satisfy the world energy consumption, integration devices have been manufactured. As a result, SCs hold a true potential to alleviate intermittence and make solar electricity available at night and in cloudy days. These systems could be of particular interest in sustainable energy storage systems.

## 6 Conclusions and Outlook

In this review, we have summarized the development of electrode materials and device configurations. For the active electrode materials, we have reviewed the development on electrode materials design for high‐performance SCs. Electrode materials with various morphology and structure play a crucial role in determining the overall capacitive behaviors including specific capacitance, rate capability, cycling life and energy density.^587^ Various types of electrode materials can be classified into three categories: (1) carbon materials, (2) metal compounds and (3) conducting polymers.

From devices perspective, new‐concept SCs have been represented including MSCs, fiber SCs and Li/Na‐ion capacitors. MSCs and fiber SCs have been developed to meet the potential applications in next‐generation roll up displays, wearable and stretchable devices. On one hand, MSCs can be integrated into flexible and stretchable chips in series or parallel which can increase the density of SCs and improve the output potential and/or current. On the other hand, fiber SCs have drawn extensive interest owing to their lightweight, flexible and wearable properties. Fiber SCs are fabricated by arranging two fiber electrodes either helically or in parallel, which are easily folded, rolled up, woven into textiles or even reshaped into other architectures.

Subsequently, Li‐/Na‐ capacitors have been developed as attractive high energy devices combining a typical battery‐type electrode with an EDLC carbon‐based electrode. The positive electrode provides the necessary power density depending on the reversible nonFaradaic charge storage mechanism. The negative electrode undergoes a Faradaic lithium/sodium ion insertion/extraction reaction. Consequently, Li‐/Na‐ion capacitors typically exhibit energy densities of around 30 Wh kg^–1^, which is three times larger than that of EDLCs. Moreover, Li‐/Na‐ capacitors display superior power densities and improved cycling stability compared with lithium ion batteries.

Recently, extensive efforts have been devoted to integrated photovoltaic cells and SCs to realize energy conversion and storage in a single unit. The integration devices are solar‐driven and self‐powered systems with good photo‐response, excellent stability and high reproducibility. Moreover, the boosting development of integration devices delivers the light‐weight and high flexibility characteristics for their potential application in portable devices. In this review, we have briefly introduced integration systems combined with photovoltaic cells and SCs. To optimize energy utilization efficiency and reduce device volume, photo‐supercapacitors without external connection have been designed due to their lightweight, high efficiency and flexibility.

In our opinion, enormous efforts have spawned the significant advancement in electrode materials design and devices construction for high‐performance SCs. The improved synthesis techniques generate optimized electrode materials with increased electroactive interfaces, which favor the charge accumulation and efficient electrolyte ions transportation. Device configurations are evolved in accordance with the rapidly growing requirement on small, thin, lightweight, and flexible electronic devices. Recent development on device configuration has paved a new way for the further applications. However, a series of challenging issues still remain for promoting the practical application of SCs.

Firstly, it is of great scientific and industrial importance to explore new types of effective electrode materials or structures to fabricate high‐performance devices with facile methods. EDLC‐based electrode materials possess high power density and excellent long‐term cycling stability but limited energy density. Pseudocapacitive electrode materials can deliver higher specific capacitance and energy density but unsatisfied power density and cycling performance. Previous reports have developed the composite materials as promising candidates with multifunctional interpenetrating nanostructured components. Enhance properties are investigated due to the combination of EDLCs and pseudocapacitive charge storage. However, complex fabrication methods hinder their wider application. The challenge is to explore new types of electrode materials or structures to fabricate high‐performance SCs by a cost‐effective technology.

Secondly, the capacitive properties of solid‐state energy devices are improved including their volumetric capacitance, energy density and power density. In this regard, further research should focus on the advancement of flexible electrodes and solid‐state electrolytes. On one hand, relatively inadequate types of materials have been explored as flexible electrodes with high mechanical flexibility. Compared to carbonaceous materials, pseudocapacitive materials are expected to apply in flexible electrodes with tailored architectures for high energy density due to their redox reaction. In addition, present preparation approaches for flexible electrodes are difficult for a large‐scale requirement. Consequently, considerable efforts are anticipated to massively develop various types of flexible electrode with satisfied electrochemical performances. Future researches lie in flexible, thin, lightweight electrode and devices to generate innovative products such as skin sensors, wearable displays, and electronic paper and so on. On the other hand, the general electrolytes are gel polymers. The limited ionic conductivities and potential window hinder the devices performances in power density and energy density, respectively. Hence, it is desirable to explore new types of solid electrolytes or improve present gel electrolytes with high ionic conductivities and wide voltage window. Moreover, the safe operation should be taken into account for their promising application in wearable electronics and smart textiles.

Thirdly, integration systems have been an interest field by incorporating SCs devices with other energy harvesting or storage devices such as piezoelectric nanogenerators, solar cells, and thermoelectric cells) and other electronic circuits.^588^ On one hand, impressive progresses have been made on the advancement of integrated systems with energy harvesting devices. However, there are still some important issues to be tackled. Previous integrated system has seldom realized full flexibility while flexible integrated systems are urgent requirement for the smart, ubiquitous electronic devices. Furthermore, optimized integration systems with high energy utilization efficiency are desired to improve the total photoelectric conversion and storage efficiency and offer a promising way for dynamic load‐levelling operations. On the other hand, the fabrications of various types of integration systems have been hopeful field to accommodate with different specific requirements such as electrochromic, self‐healing systems and so on.
